# Transcriptional network analysis of PTEN‐protein‐deficient prostate tumors reveals robust stromal reprogramming and signs of senescent paracrine communication

**DOI:** 10.1002/1878-0261.70164

**Published:** 2025-11-17

**Authors:** Ivana Rondon‐Lorefice, Jose I. Lopez, Aitziber Ugalde‐Olano, Maite Zufiaurre, Ianire Astobiza, Natalia Martin‐Martin, Laura Bozal‐Basterra, Saioa Garcia‐Longarte, Amaia Zabala‐Letona, Sofia Rey, Aida Santos‐Martin, Miguel Unda, Ana Loizaga‐Iriarte, Mariona Graupera, Paolo Nuciforo, Arkaitz Carracedo, Isabel Mendizabal

**Affiliations:** ^1^ Center for Cooperative Research in Biosciences (CIC bioGUNE), Basque Research and Technology Alliance (BRTA) Derio Spain; ^2^ Biocruces Bizkaia Health Research Institute Leioa Spain; ^3^ Centro de Investigación Biomédica en Red de Cáncer (CIBERONC) Madrid Spain; ^4^ Department of Pathology Basurto University Hospital Bilbao Spain; ^5^ Traslational Prostate Cancer Research Lab, CIC bioGUNE‐Basurto Biocruces Bizkaia Health Research Institute Bilbao Spain; ^6^ Department of Urology Basurto University Hospital Bilbao Spain; ^7^ Endothelial Pathobiology and Microenvironment Group, Josep Carreras Leukemia Research Institute (IJC) Barcelona Spain; ^8^ ICREA Barcelona Spain; ^9^ Molecular Oncology Group, Vall d'Hebron Institute of Oncology (VHIO) Barcelona Spain; ^10^ Ikerbasque, Basque Foundation for Science Bilbao Spain; ^11^ Biochemistry and Molecular Biology Department University of the Basque Country (UPV/EHU) Bilbao Spain

**Keywords:** prostate cancer, PTEN protein loss, stratification, stromal remodeling

## Abstract

Among the extensive genomic alterations in prostate cancer, phosphatase and tensin homolog (*PTEN*) deletion stands out as one of the most consistently observed events. *PTEN* loss in prostate tumors is primarily associated with cancer‐cell proliferation and survival through the activation of the phosphoinositide 3‐kinase (PI3K)—protein kinase B (AKT)—mechanistic target of rapamycin (mTOR) (PI3K–AKT–mTOR) signaling pathway. However, the use of PTEN as a robust biomarker in clinical practice is hampered by its complex epigenetic, transcriptional and post‐translational regulation. *In situ* protein assessment by immunohistochemistry (IHC) captures PTEN protein status, but it does not report on associated tumor microenvironment remodeling. Here, we undertook an approach that combined PTEN immunoreactivity analysis with high‐throughput transcriptional analysis to gain insights into the downstream functional effects of PTEN protein loss in primary tumors. Our extensive bioinformatic analyses highlighted stromal remodeling as a prominent cancer cell‐extrinsic process associated with PTEN loss. By extending our transcriptomic computational strategy to *Pten* loss‐driven murine prostate cancer, we validated the causal role of *Pten* in the stromal reaction observed in clinical specimens. Mechanistically, we provide experimental evidence for the activation of a paracrine program that encompasses enhanced transforming growth factor beta (TGF‐β) signaling and that is compatible with the secretome of *PTEN*‐deficient senescent cancer cells. Finally, our findings enable the sub‐stratification of tumors with PTEN loss based on their senescence‐associated stroma remodeling program to distinguish indolent from aggressive cases. Our study provides relevant biological context to the cellular and molecular alterations unleashed upon PTEN protein loss in prostate cancer.

## Graphical abstract

Combining PTEN protein assessment and transcriptomic profiling of prostate tumors, we uncovered a network enriched in senescence and extracellular matrix (ECM) programs associated with PTEN loss and conserved in a mouse model. We show that PTEN‐deficient cells trigger paracrine remodeling of the surrounding stroma and this information could help stratify PTEN‐negative tumors according to clinical aggressiveness.

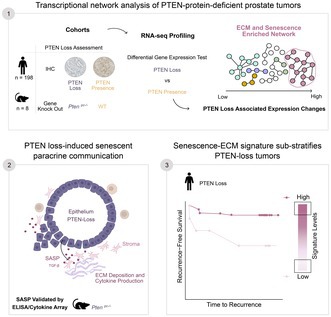

**Abbreviations**
DEG: differentially expressed gene
ECM: extracellular matrix
ELISA: enzyme‐linked immunosorbent assay
FDR: false discovery rate
FFPE: formalin‐fixed paraffin‐embedded
GSEA: gene set enrichment analysis
HR: hazard ratio
H‐score: histochemical score
IHC: immunohistochemistry
LR: likelihood ratio
PI3K–AKT–mTOR: phosphoinositide 3‐kinase–protein kinase B–mechanistic target of rapamycin
SASP: senescence‐associated secretory phenotype
TCGA: the cancer genome atlas
TGF‐β: transforming growth factor beta
TMA: tissue microarrays
UMAP: uniform manifold approximation and projection
WGCNA: Weighted Gene Correlation Network Analyses

## 1 Introduction

Phosphatase and tensin homolog deleted in chromosome 10 (*PTEN*) is a well‐established tumor suppressor gene frequently disrupted in cancer. Germline and somatic mutations in *PTEN* have been associated with an increased risk for various types of malignancies. In prostate cancer, *PTEN* is among the most frequently altered genes in the epithelial gland, with 15–20% of patients presenting somatic genetic aberrations in primary tumors and up to 50% in patients with advanced disease [1, 2, 3].

Loss of PTEN function results in the activation of the oncogenic PI3K signaling pathway. In prostate cancer, PTEN loss is associated with alterations in cell proliferation, migration, survival, metabolism, and genomic stability, among others [4]. In addition to these well‐characterized cancer‐cell‐autonomous processes, recent studies have established critical roles of PTEN in the reprogramming of the tumor microenvironment, promoting immunosuppressive and pro‐tumorigenic stroma in the prostate [2, 5]. Yet, it is unclear whether those changes in the tumor microenvironment can be exploited for patient stratification.

The characterization of PTEN status in prostate cancer predominantly focuses on genomic loss. In multiple clinical cohorts, deletion of *PTEN* is strongly associated with a higher Gleason score [6] and poorer prognosis [7, 8]. Yet, the assessment of *PTEN* gene status has not been translated into a robust prognostic or therapeutic biomarker in clinical practice. This is possibly due to the highly complex regulation of its functions. *PTEN* regulation by promoter DNA methylation and noncoding RNAs, such as miR‐106a [9], miR‐26a [10] and PlncRNA‐1 [11] or competitive endogenous RNAs [12, 13], has been reported, as well as several post‐translational modifications, including phosphorylation [14] and ubiquitination [15]. Hence, genomic status might not fully capture the molecular and pathological consequences of the loss of PTEN activity in prostate cancer.

Assessing PTEN protein levels in tissues can be more relevant as a clinical marker to develop new strategies for diagnosis and treatment. For example, PTEN protein status could guide stratification in clinical trials for treatment drugs, such as PI3K or mTOR inhibitors. In addition, previous studies have shown that PTEN loss is frequently focal and subclonal in prostate tumors [16, 17]. In this context, *in situ* methodologies such as immunohistochemistry (IHC) can be preferable to spatial‐agnostic assays. Yet, IHC alone does not report on the downstream reprogramming of the tumor microenvironment that may influence patient outcomes.

Here, we adopted a data‐driven strategy to chart the molecular consequences of PTEN protein loss in human prostate tumors. By combining spatially resolved PTEN IHC in a cohort of 197 patients with computational analyses of bulk and single‐cell RNA sequencing, we define a senescence‐associated paracrine stroma remodeling program activated in tumors with PTEN protein loss. We experimentally validate this program in mouse models and demonstrate its prognostic value to refine PTEN‐based stratification. This framework, integrating PTEN immunoreactivity analysis, transcriptomic studies, mouse genetics, and clinical data, provides an innovative biological perspective of PTEN function in prostate cancer with clinical implications.

## 2 Materials and methods

### 2.1 Tissue collection

We collected 197 tissue specimens from localized primary prostate tumors from Basurto University Hospital in Bilbao (Spain, Table S1). The tissues were derived from prostatectomies following previous methods [18] and were preserved in formalin‐fixed paraffin‐embedded (FFPE) blocks. Sample collection was coordinated by the Basque Biobank and was performed between September 2011 and November 2013. The study was conducted in accordance with the ethical guidelines and approved protocols CEIC‐E 11–12, 14–14, and 19–20 and in agreement with the standards set by the Declaration of Helsinki. The experiments were undertaken with the understanding and written consent of each subject.

See Table 1 for detailed information on the clinical and pathological characteristics of the cohort. Tumor‐rich regions were selected for RNA extraction by the pathologist.

**Table 1:** Clinical information of the patient cohort described in this study.

Characteristics	Primary tumors (197)
Age	
Median	64
Mean	62.8
Standard deviation	6.2
Min–max	44–76
BMI (kg·m^−2^)	
Median	26.7
Mean	26.8
Standard deviation	3
Min–max	19.3–36.67
PSA at diagnosis (ng·mL^−1^)	
Median	6.38
Mean	7.71
< 4	2 (1%)
4–10	160 (81%)
> 10	31 (15%)
NAs	4 (3%)
ISUP grade	
1	16 (8%)
2	128 (65%)
3	31 (16%)
4	4 (2%)
5	18 (9%)
Ethnicity	
White	197 (100%)
Follow‐up time after surgery (months)	
Median	90.6
Mean	73.7
Standard deviation	33.1
Min–max	0.46–109.4
NAs	9 (4.6%)

### 2.2 Immunohistochemical staining of PTEN

The full cohort was represented in 10 tissue microarrays (TMA), in which each patient was represented with two tumor cell‐rich cores of 1 mm in diameter. The TMA slides were dewaxed at 72 °C, incubated with an anti‐PTEN rabbit monoclonal primary antibody (clone 138G6, 1/100 diluted, #9559 Cell Signaling Technology, Danvers, MA, USA) for 60 min at 36 °C and detected using Ultraview DAB IHC detection Kit (cat. no.: 760‐500) on the VENTANA BenchMark ULTRA automated staining platform. The slides were mounted and digitalized at 20× using a slide scanner (NanoZoomer 2.OHT; Hamamatsu Photonics, Shizuoka prefecture, Japan).

### 2.3 PTEN immunohistochemical scoring

**Fig. 1:**
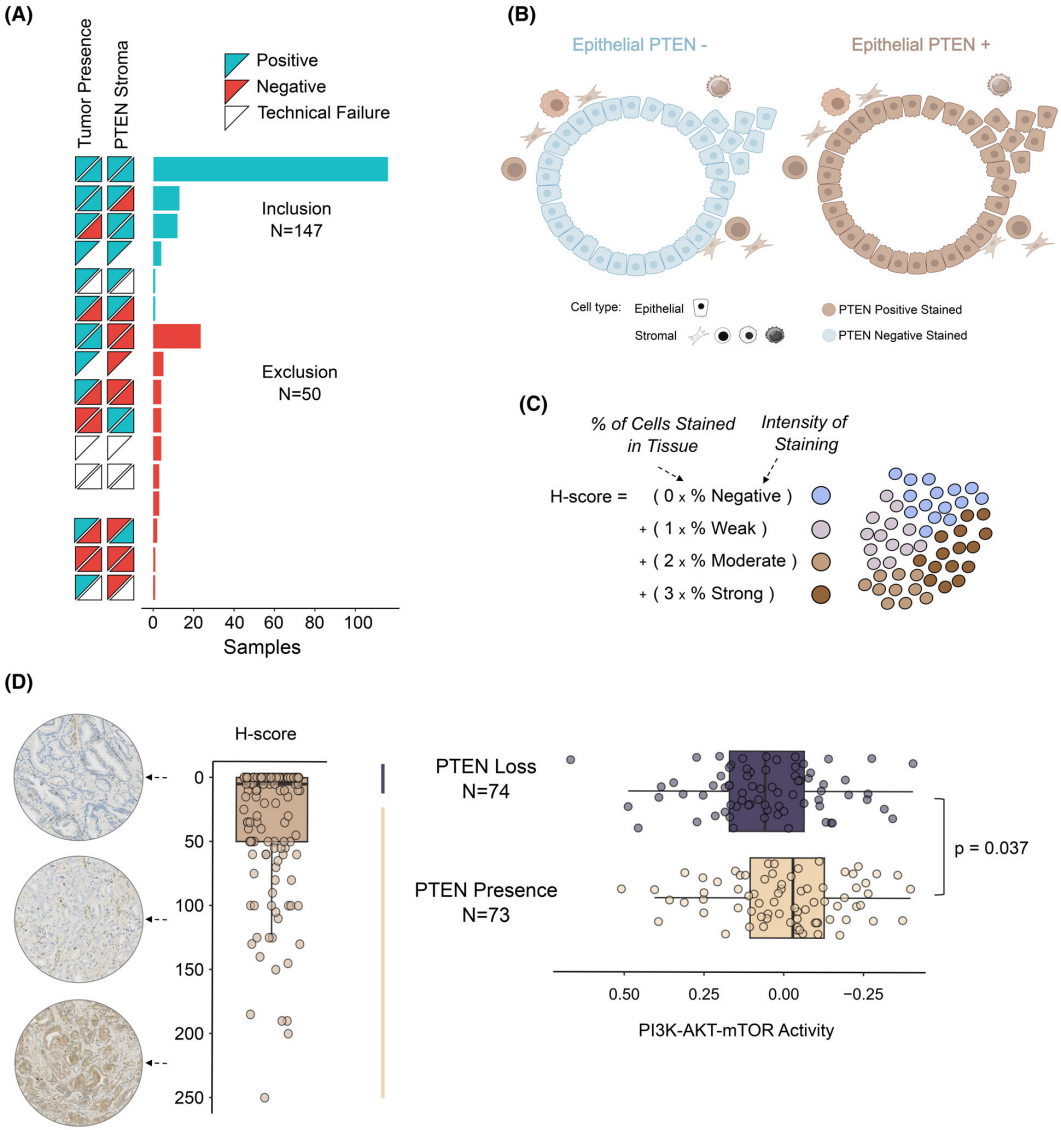
PTEN protein loss assessment in clinical prostate cancer specimens. (A) Tissue microarrays from prostate specimens obtained from prostatectomies were employed to assess the status of PTEN by immunohistochemistry (IHC). Each row represents a case type, grouping samples based on tumor presence and stromal PTEN staining across evaluated cores. Individual cores are represented by triangles. Tumor presence is indicated as positive (cyan triangles), negative (red triangles), and white for technical failure in the ‘Tumor Presence’ column. Stroma PTEN staining is shown similarly: cyan for positive, red for negative and white for undetermined staining. The bar plot on the right indicates the number of cases included (*N* = 147) based on core‐level evaluations. Samples that failed to present at least one core with proper counterstaining, presence of tumoral areas, or showed negative or invalid staining for PTEN in the stromal cells were discarded from downstream analysis. (B) Graphical representation of the stromal staining used as quality control to confirm epithelial PTEN signal loss (PTEN ‐) or presence (PTEN+). In the diagram, brown represents PTEN IHC‐positive cells, and blue represents PTEN IHC‐negative cells. (C) The H‐score was computed to measure the staining of each sample based on the number of cells stained in the tissue and their intensity. (D) Left panel: Distribution of H‐scores across the 147 prostate cancer samples. Arrows indicate representative images from the IHC assay. High H‐scores correspond to strong PTEN staining (brown), while low H‐scores indicate the absence of PTEN protein (blue). Right panel: The cut threshold at H‐score of 0 was employed to categorize the samples into PTEN‐loss and PTEN‐presence groups, which showed significant differences in PI3K‐AKT–mTOR signature. Boxplots indicate the median (center line), interquartile range (box), and whiskers extending to the most extreme data points within the interquartile range. Two‐tailed Wilcoxon's tests were used to calculate *P*‐values.

Following digitalization, prostate tissues were assessed by a board‐certified pathologist at the Cruces Hospital at a microscope. As technical controls, we evaluated the positive counterstain with hematoxylin, the presence of tumoral regions, and PTEN positive staining in the stroma per core. A total of 50 samples did not pass these criteria in at least one core and were excluded from downstream analyses (Fig. 1A and Table S2).

With the 147 samples that passed the quality controls, the percentage of cells exhibiting negative, weak, moderate, and intense staining in the epithelia was then utilized to calculate the H‐score, calculated as follows (where × means multiplication):

$$H‐\text{score}=\begin{array}{l} 3\times \left(\%\text{strongly staining cells}\right)\\ +2\times \left(\%\text{moderately staining cells}\right)\\ +1\times \left(\%\text{weakly staining cells}\right) \end{array}$$

When two cores are available per patient, the mean H‐score is reported.

### 2.4 RNA‐seq library generation and data processing of human specimens

RNA was extracted from paraffin‐embedded tissue sections using a standardized protocol. Following deparaffinization, enzymatic digestion and heat treatments were applied to facilitate tissue breakdown and nucleic acid release. The RNA was purified using silica‐based column technology, incorporating DNase treatment to remove contaminating DNA. Eluted RNA was assessed for integrity and concentration using spectrophotometry, gel electrophoresis, and capillary electrophoresis systems. Samples were stored at −80 °C. Sequencing libraries were prepared following the SMARTer Stranded Total RNA‐Seq Kit v2 – Pico Input Mammalian protocol (cat. no.: 634411; Takara Bio, Kusatsu, Japan), as detailed in the SMARTer Stranded Total RNA‐Seq Kit v2—Pico Input Mammalian User Manual (Rev. 050619). The libraries were sequenced using the NovaSeq 6000 platform with paired‐end reads of 150 base pairs in length (average 63 million reads per sample).

Adaptor sequences specified by the Illumina TruSeq RNA kit were removed using cutadapt [19] (v4.8) with the following command: cutadapt ‐a AGATCGGAA GAGCACACGTCTGAACTCCAGTCA ‐A AGATCGGAAGAGCGTCGTGTAGGGAAAGAGTGT. Additionally, three nucleotides were trimmed from the R1 and R2 strands, respectively, as specified by the library preparation protocol using the command: cutadapt ‐q 10 ‐‐minimum‐length 20 –u 3 ‐U 3. The quality of the reads was verified using FastQC [20] (v0.12.1).

Reads were mapped to the reference human genome GRCh38 using the Spliced Transcripts Alignment to a Reference (STAR) [21] v2.7.10 a with the following parameters: ‐‐outFilterMultimapNmax 1 ‐‐outReadsUnmapped Fastx ‐‐outSAMtype BAM SortedByCoordinate ‐‐twopassMode Basic ‐‐limitBAMsortRAM 2000000000 ‐‐quantMode TranscriptomeSAM GeneCounts. The gene annotation file (GTF) for human was obtained from the Ensembl [22] release v94.

### 2.5 RNA‐seq library generation and data processing of murine data

RNA was extracted from fresh tissues and sequenced using the Illumina HiSeq4000 platform with paired‐end reads of 100 base pairs in length (average 66 million reads per sample). Sequencing libraries were prepared following the ‘TruSeq Stranded Total RNA Human/Mouse/Rat’ kit (cat. no.: RS‐122‐2201; Illumina Inc., San Diego, CA, USA), using ‘TruSeq Stranded Total RNA Sample Prep‐guide (Part#15031048Rev.E)’. The quality of the reads was verified using FastQC [20] (v0.12.1).

Reads were mapped to the reference mouse genome GRCm39 using the Spliced Transcripts Alignment to a Reference (STAR) [21] v2.7.10 a with the following parameters: ‐‐outFilterMultimapNmax 1 ‐‐outReadsUnmapped Fastx ‐‐outSAMtype BAM SortedByCoordinate ‐‐twopassMode Basic ‐‐limitBAMsortRAM 2000000000 ‐‐quantMode TranscriptomeSAM GeneCounts. The corresponding gene annotation file (GTF) was obtained from the Ensembl [22] release v110.

### 2.6 Differential expression and Weighted Gene Correlation Network Analyses (WGCNA)

RNA‐Seq data were normalized using the TMM method using edgeR [23] (v4.0.16) and limma voom [24] (v3.58.1) was used to identify the differentially expressed genes (DEGs) between individuals with PTEN protein loss vs presence condition. In the case of the human dataset, we considered age and integrity index DV200 index (percentage of RNA fragments > 200 nucleotides) [25] with z‐score transformation. Differentially expressed genes were identified using a threshold of |log_2_(FC)| > 0 and false discovery rate (FDR) < 0.05; and |log_2_(FC)| > 1 and FDR < 0.05 for human and mouse datasets, respectively.

We performed gene co‐expression network analysis using Weighted Gene Correlation Network Analyses (WGCNA) [26] (v1.72.1) in R. Briefly, in WGCNA, the construction of the network is based on soft thresholding the correlation coefficient, to which the co‐expression similarity is raised to calculate the adjacency. In the case of human data, we filtered out genes with less than five counts in more than 70% of the samples; for the mouse data, we filtered out genes with less than five counts in more than 90% of the samples. We subsequently applied variance‐stabilization transformation to the gene expression matrices for both human and mouse using DESeq2 [27] (v1.42.1).

To select the correct parameter for network construction, we inferred the soft‐threshold power by approximating our network to a scale‐free topology using a signed Spearman correlation. For the human dataset, we selected a power of 14 based on the results obtained with the function ScaleFreeTopology. Then, we ran the function blockwiseModule passing through the following parameters maxBlockSize = 25000, corType = ‘bicor’, maxPOutliers = 0.1, pearsonFallback = ‘individual’, networkType = ‘signed’, deepSplit = 1, mergeCutHeight = 0.1. In the mouse dataset the parameters passed to the blockwiseModule function were the following: maxBlockSize = 20000, corType = ‘bicor’, maxPOutliers = 0.1, power = 18, networkType = ‘signed’, deepSplit = 2, mergeCutHeight = 0.25.

To intersect human and mouse modules, conversion of mouse gene names to human was performed using the gene homology data from Ensembl [22].

### 2.7 Functional enrichment analyses

We used Gene Set Enrichment Analysis (GSEA) [28] (v4.3.2) after applying the normalization method ‘median of ratios’ with DESeq2 [27] (v1.42.1). We identified the enriched pathways in PTEN protein loss vs presence in the following gene sets databases for Human Collection (MSigDB): h.all.v2023, c5.go.bp.v2023, c5.go.cc.v2023, c5.go.mf.v2023, c2.cp.reactome.v2023. We used the permutation type by ‘gene_set’ and performed 1000 permutations.

Overrepresentation analyses were carried out with the ‘gprofiler’ package [29] (v0.7.0). For module enrichment analysis, the background was tuned by all genes evaluated in the module construction. The following parameters were selected: organism = ‘hsapiens’ or ‘mmusculus’, multi_query = FALSE, significant = TRUE, exclude_iea = FALSE, measure_underrepresentation = FALSE, evcodes = TRUE, user_threshold = 0.05, correction_method = ‘fdr’, domain_scope = ‘custom’, numeric_ns = ‘‘, sources = NULL, as_short_link = FALSE.

### 2.8 Signature scoring

To compute signature scorings, we first subsetted the normalized log_2_ expression matrix to include only the genes in each signature. We then scaled the expression values using z‐scores across all samples and calculated the average z‐scored expression of the signature genes for each sample.

### 2.9 *In silico* deconvolution analyses

We estimated the purity (cancer‐cell proportion) per sample by ESTIMATE [30] through applying the tidyestimate R package (v1.1.0) based on the GSEA of stromal and immune gene sets. For extensive cell‐type deconvolution across 64 cell types, we ran xCell [31] (v1.1.0) using transcripts per million (TPM) normalization. We further refined our analysis by estimating cell‐type proportions specific to prostate tissue using MuSiC [32] (v1.0.0).

### 2.10 Single‐cell analysis

To evaluate the cell‐type specificity of the detected modules, we utilized single‐cell RNA‐Seq data (10X Genomics) from GEO (accession ID: GSE141445) [33]. Specifically, we selected eight localized prostate cancer samples from the processed count matrix, which had mitochondrial genes filtered and quality control (QC) steps completed by the original authors. The data were normalized using SCTransform with v2 regularization implemented in Seurat [34] (v4.2.1). For sample integration, 3000 anchor genes were identified and used with canonical correlation analysis (CCA) for dimensionality reduction. Subsequently, linear dimensionality reduction and uniform manifold approximation and projection (UMAP) were performed based on the top 16 principal components (PCs). Clustering was carried out with a resolution parameter set to 0.8. Cluster‐specific markers were identified using Seurat's FindAllMarkers function, and cell types were annotated based on well‐established markers. Finally, the projection of modules into the single‐cell data was done with UCell [35] (v2.6.2), which is a method that measures how strongly a given set of genes is expressed in each single cell, helping to identify cells with specific functional characteristics.

### 2.11 Gene fusion analysis

Gene fusions were inferred from transcriptomic data using STAR‐Fusion [36] (v1.7.0), which identifies candidate fusion transcripts by analyzing chimeric junctions from RNA‐Seq data aligned with the STAR aligner [21] (v2.7.11a) against the human reference genome (GRCh38) and using the CTAT_HumanFusionLib.v0.1.0.dat reference library. STAR‐Fusion outputs preliminary fusion candidates based on split and discordant read evidence. To validate and refine these predictions, we utilized FusionInspector, which reconstructs fusion transcript sequences and re‐aligns reads to synthetic fusion contigs. This step provides enhanced confidence metrics, including junction‐spanning read counts and expression estimates (FFPM: fusion fragments per million reads). Following fusion detection, we applied custom filtering criteria to retain high‐confidence events and reduce artifacts. Specifically, we retained only fusions meeting the following thresholds FFPM ≥ 0.1 and JunctionReadCount ≥ 1. To further increase specificity, we excluded candidate fusions involving likely artifacts, such as pseudogenes and noncoding RNAs (e.g., gene names matching ‘RNA5SP’ or starting with ‘AC’). We also filtered out noncanonical splicing events by excluding fusions with ‘INCL_NON_REF_SPLICE’ in the SpliceType field. Only fusions passing all criteria were included in downstream analyses. For each sample and fusion pair, multiple reported isoforms or breakpoint variants (e.g., repeated TMPRSS2‐ERG calls in a single sample) were collapsed into a single event to facilitate interpretation. In these cases, we aggregated read support by summing the JunctionReadCount and SpanningFragCount, and averaged expression metrics (est_J, est_S) across isoforms.

### 2.12 TCGA data processing

To analyze RNA‐Seq data from prostate cancer specimens, we used publicly available TCGA data accessed via the Bioconductor Recall package v1.28.0. The study‐specific RangedSummarizedExperiment object was downloaded using the download_study() function. Raw count data were scaled using scale_counts() to account for library size and sequencing depth. We then filtered the dataset to include only primary tumor samples from patients diagnosed with Prostate Adenocarcinoma, excluding duplicates based on unique submitter IDs. Low‐expressed genes were removed by retaining those with counts > 5 in more than 50% of the samples. Gene annotations were retrieved from the rowData() of the RangedSummarizedExperiment and merged with external genome annotation data (GRCh38) to map Ensembl IDs to gene symbols and lengths.

To define *PTEN* genomic status, we integrated GISTIC2‐processed copy number alteration (CNA) data from TCGA. *PTEN* homozygous deletion (HOM loss) was assigned to samples with a GISTIC score of −2, indicating a complete loss of both alleles. Samples with a GISTIC score of 0 were considered *PTEN*‐intact. Samples with heterozygous loss (−1) were excluded from binary comparisons to ensure clearer separation between fully deleted and intact cases.

### 2.13 Protein extraction from murine anterior prostate tissue

Prostate epithelium‐specific deletion Pten‐knockout (C57/BL6/129sv; Pb‐Cre4; Pten lox/lox) model was kindly provided by Dr. Pandolfi. All mouse experiments were carried out following the ethical guidelines established by the Biosafety and Welfare Committee at CIC bioGUNE (Spanish acronym for center for cooperative research in Biosciences). The procedures employed were carried out following the recommendations from AAALAC (Association for Assessment and Accreditation of Laboratory Animal Care). Mice were maintained in a controlled environment, with standard 12:12 light:dark cycles, 30–50% humidity, and controlled temperature at 22 ± 2 °C. Diet and water were provided *ad libitum*. At the experimental endpoint, mice were sacrificed by CO_2_ inhalation followed by cervical dislocation. The study was approved by the Bioethics and Welfare Committee under the code P‐CBBA‐0121.

Anterior prostates were collected from three 6‐month‐old *Pten* lox/lox and three wild‐type (WT) male mice and homogenized in 500 μL of PBS supplemented with a protease inhibitor cocktail (04693159001; Roche, Basel, Switzerland) using six 2.8 mm ceramic beads in a Precellys tissue homogenizer (Bertin Technologies, Frankfurt am Main, Germany) at 3000 r.p.m. for 2 cycles of 30 s each, with a 5‐s break between cycles. The homogenates were then lysed with 1% Triton X‐100 final concentration, vortexed twice for 30 s, and stored at −80 °C overnight. Then, samples were thawed on ice and the Pierce™ BCA Protein Assay Kit (Cat: 23227, Thermo Scientific, Waltham, MA, USA) was performed. The same protein extracts were used for both cytokine array and ELISA experiments.

### 2.14 Cytokine array experiments

Two milligrams of total protein per sample were analyzed using the Proteome Profiler Mouse XL Cytokine Array (ARY028; R&D Systems) according to the manufacturer's instructions. Briefly, array membranes were blocked for 1 h at room temperature with Array Buffer 6. After blocking, 200 μg of total protein was incubated in a final volume of 1.5 mL with Array Buffer 4 and 6, overnight at 4 °C on a rocking platform shaker. After three washes using 1× Wash Buffer for 10 min each, the membranes were incubated with 1.5 mL of Detection Antibody Cocktail for 1 h at room temperature, followed by three washes and Streptavidin‐HRP incubation for 30 min on a rocking platform shaker. Chemiluminescence signals were developed using Chemi Reagents 1 and 2 and images were acquired using an iBright imaging system. Cytokine spot intensities were quantified using the ROI1 click plugin on imagej [37] v1.54p.

### 2.15 TGF‐β1 and TGF‐β2 protein level quantification by ELISA

The Human/Mouse/Rat/Porcine/Canine TGF‐β1 and TGF‐β2 Quantikine ELISA Kits (R&D Systems, Abingdon, UK; DB100C and MB200, respectively) were used to quantify the levels of active TGF‐β1 and TGF‐β2 in tissue homogenates, following the manufacturer's protocol for cell lysates (as specified in the TGF‐β2 protocol). Briefly, 20 or 40 μg (for TGF‐β1 or TGF‐β2, respectively) of total protein per sample was used in a final volume of 100 μL. Samples were activated by incubation for 10 min at room temperature with 20 or 50 μL of 1 N HCl (for TGF‐β1 or TGF‐β2, respectively) and subsequently neutralized with an equal volume of 1.2 N NaOH/0.5 m HEPES. Fifty microliters of Assay Diluent RD1‐21 or RD1‐98 (for TGF‐β1 or TGF‐β2, respectively) were added to each well on the ELISA plate, followed by 50 μL of standard, control, or activated sample, all in duplicates. Plates were then covered and incubated for 2 h at room temperature: on the benchtop for TGF‐β1, or on a horizontal orbital microplate shaker set to 500 rpm for TGF‐β2. After four washes with 1× Wash Buffer, 100 μL of TGF‐β1 or TGF‐β2 conjugate (as appropriate) were added per well. Plates were incubated for 2 h under the same respective conditions (benchtop or shaker), followed by four additional washes. Subsequently, 100 μL of Substrate Solution were added to each well and incubated for 30 min at room temperature, protected from light. The reaction was stopped by adding 100 μL of Stop Solution per well. Optical density was measured using a BioTek Epoch microplate spectrophotometer at 450 nm, with wavelength correction set to 540 nm.

## 3 Results

### 3.1 PTEN protein is extensively lost in prostate primary tumors

To assess the loss of PTEN protein in prostate tumors, we assembled a tissue microarray from a cohort of 197 patients from Basurto University Hospital with newly diagnosed disease (Table 1 and Table S1 for detailed clinical and pathological characteristics of the cohort). We performed IHC in FFPE blocks obtained from prostatectomies. For most patients, two cylindrical sections (cores) per individual were available. We evaluated each core and assessed PTEN immunoreactivity using a clinical grade IHC assay (ISO15189‐accredited) as previously described [38]. Using hematoxylin counterstaining, pathologists localized tumoral areas and evaluated PTEN staining in epithelial and stromal regions. To control false negatives in the analysis, we evaluated PTEN separately in the epithelium and the stroma. Whereas genetic and epigenetic alterations could result in loss of PTEN immunoreactivity in the epithelium, we established that loss of signal in the stroma would most probably be due to problems with sample or epitope preservation. Therefore, we established stromal PTEN positivity as a sample quality control for assessing PTEN immunoreactivity in the epithelium.

Out of 197 samples, 147 presented at least one tumor core with positive stromal PTEN staining (Fig. 1A). Fifty specimens were excluded from further analysis due to tumor absence, negative stromal PTEN immunoreactivity or staining failure (Fig. 1A,B and Table S2). For tumoral areas with positive stroma staining, we calculated the Histochemical score (H‐score) by multiplying the percentage of epithelial cells at each PTEN staining intensity (negative, weak, moderate, and intense) by a corresponding weight (0–3) and summing the values (Fig. 1C). The H‐score is a semi‐quantitative metric that integrates staining intensity and positive cell proportion into a single continuous value [39] and is widely used in oncology to provide a more nuanced assessment than binary scoring [40]. Immunostaining values for PTEN in the primary prostate tumors showed a skewed distribution toward lower H‐scores (median = 5, mean = 32.69, s.d. = 50.86, Fig. 1D left panel and Table S1).

### 3.2 PTEN protein loss associates with extracellular matrix processes

To understand the impact of PTEN protein loss on the transcriptomic landscape of prostate tumors, we combined the immunoreactivity of the protein with RNA sequencing on this same cohort. Considering the canonical activation of the PI3K‐mTOR pathway upon PTEN loss [4, 41], we leveraged a PI3K‐AKT–mTOR activity signature comprised of 105 genes [42] and obtained a binary classification of PTEN immunoreactivity (i.e., PTEN protein loss vs. PTEN protein presence). We monitored the statistical differences in the mean expression of PI3K‐AKT–mTOR activity between PTEN‐loss and PTEN‐presence groups at various thresholds of the H‐score (Fig. S1). We identified that an H‐score of 0 showed the best discriminative power, roughly distributing half of the samples per category (Fig. 1D right panel and Table S1). Of note, *PTEN* mRNA levels did not vary according to PTEN protein status (Fig. S1). These results suggest that PTEN protein detection by IHC is a good proxy of PI3K‐AKT–mTOR activity while *PTEN* mRNA levels may not fully capture the complexity of post‐transcriptional regulation.

**Fig. 2:**
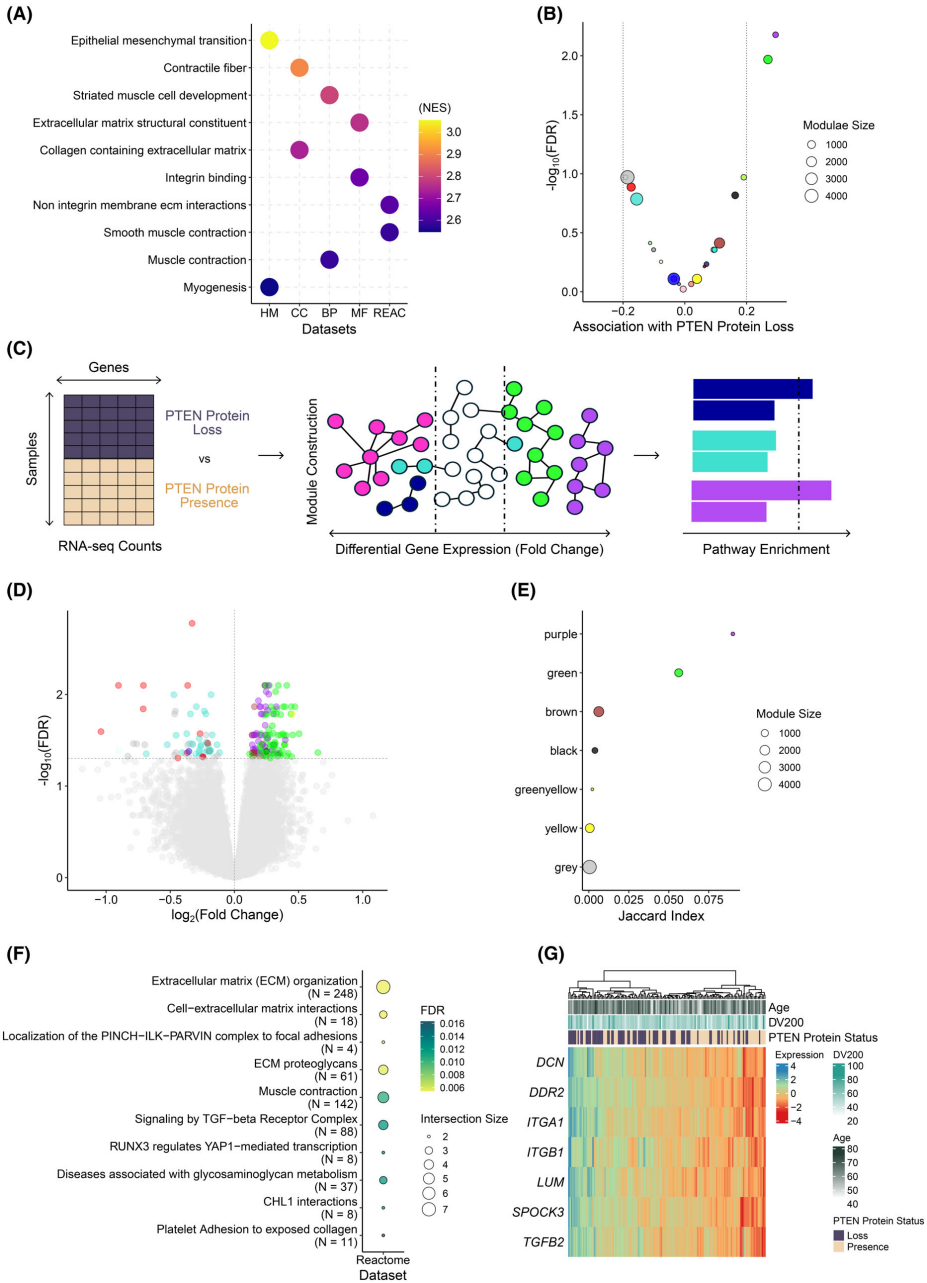
PTEN protein loss is associated with extracellular matrix (ECM) processes in prostate primary tumors. (A) Gene Set Enrichment Analysis (GSEA) comparing PTEN‐loss versus PTEN‐presence tumors, showing significant enrichment of functional terms related to ECM organization. Enrichment analyses were performed across Gene Ontology (GO) categories [Biological Process (BP), Cellular Component (CC), and Molecular Function (MF)], Reactome pathways (REAC), and Hallmark (HM) gene sets from MSigDB. Normalized enrichment score (NES); all terms have a false discovery rate (FDR) < 0.001. (B) Volcano plot showing the results of the association (Pearson's *r*) of each Weighted Gene Correlation Network Analyses (WGCNA) module with the PTEN protein status. Dot size represents the module size. Dashed lines represent statistical significance thresholds |Pearson's *r*| > 0.2 and FDR < 0.05. Colors indicate different gene co‐expression modules. The green and purple modules show the strongest positive correlations with PTEN loss. (C) Illustration of the workflow that combines co‐expressed gene module identification by WGCNA with differential gene expression and downstream functional enrichment annotation. (D) Volcano plot of differential gene expression analysis for PTEN loss vs PTEN presence. The dots are colored by WGCNA module membership. Dashed lines represent statistical significance thresholds |Pearson's *r*| > 0.2 and FDR < 0.05. (E) Intersection between differentially expressed genes (DEGs) and WGCNA modules measured by Jaccard Index. Colors indicate different gene co‐expression modules. Green and purple modules present the highest overlap with DEGs. (F) Reactome pathway enrichment for DEGs in the green module, highlighting overrepresentation of ECM related pathways. (G). Heatmap of z‐scored expression for seven DEGs from the green module involved in the ‘*Extracellular matrix organization*’ pathway.

To obtain a pathway‐centered view of differential expression patterns associated with PTEN loss, we used GSEA. Interestingly, stromal‐related functions were highly enriched, including extracellular matrix (ECM)‐related processes (Fig. 2A and Table S3). We next employed an unsupervised network‐based approach that does not rely on predefined gene categories or pathways. Weighted Gene Correlation Network Analyses [26] defines groups of genes (‘*modules*’) characterized by similar expression patterns that potentially participate in specific biological pathways in a coordinated fashion. Out of the 22 modules identified, the modules labeled ‘*purple*’ and ‘*green*’, exhibited the highest association with PTEN protein status (based on the H‐score = 0 threshold for PTEN loss; Pearson's *r* > 0.20, FDR < 0.05, Figs 2B, S2 and Tables S4, S5).

We hypothesized that the genes showing significant differential expression at both the individual gene and network levels represent the subset with the highest potential to inform about the altered molecular landscape resulting from PTEN protein loss (Fig. 2C). Gene‐by‐gene differential gene expression analyses (203 significant genes, |log_2_(fold change)| > 0, FDR < 0.05) showed that the top overexpressed genes in tumors with PTEN protein loss were enriched in the purple and green upregulated modules (Fig. 2D,E, Table S6). Functional enrichment of differentially expressed genes in the green module exhibited significant overrepresentation of ECM‐related functions (Fig. 2F,G, and Table S7). Instead, the purple module exhibited a notably higher enrichment in proliferative characteristics associated with cancer cells, such as the ‘*Activation of receptor‐tyrosine kinases*’ and ‘*PI3K signaling through fibroblast growth factor receptors*’ (Fig. S3). The information derived from the purple module is consistent with our threshold selection based on PI3K‐AKT–mTOR activity signature and aligns with biological processes associated with PTEN loss described in the literature [4]. Consequently, we chose to focus on the green module to explore the less understood roles of PTEN protein loss in the tumor microenvironment.

### 3.3 PTEN protein loss associates with stromal remodeling

**Fig. 3:**
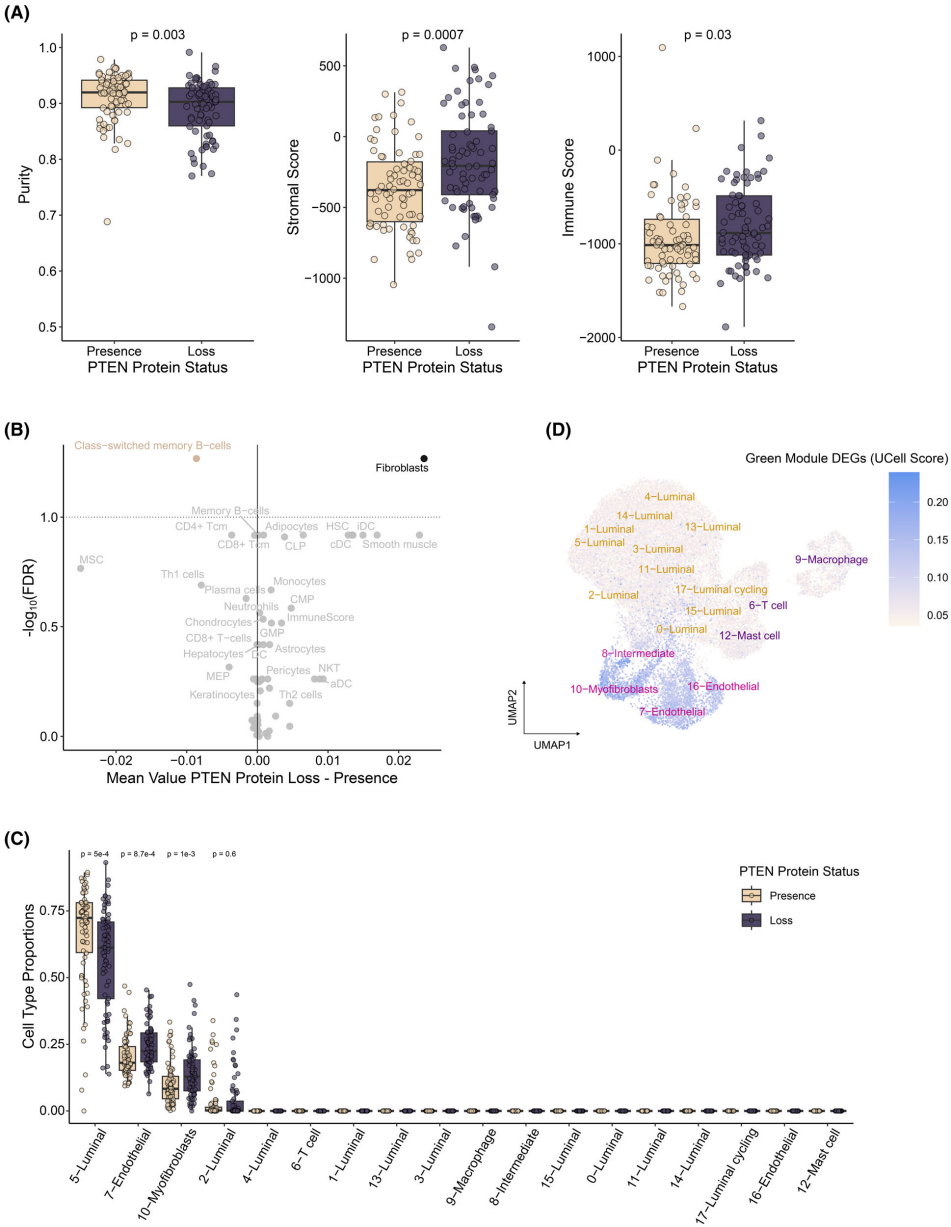
Increase in stromal composition observed with PTEN protein loss in prostate cancer tumors. (A) Cellular decomposition analysis showing the purity (the percentage of epithelial cells in the tumor) and the estimates for stromal and immune abundances using ESTIMATE. Boxplots indicate the median (center line), interquartile range (box), and whiskers extending to the most extreme data points within the interquartile range. Two‐tailed Wilcoxon's tests were used to calculate *P*‐values. (B) Detailed cell‐type decomposition shows a significant enrichment of fibroblasts at primary tumors without PTEN protein expression using xCell. The volcano plot shows the differences in mean cell‐type composition between PTEN protein loss vs presence, along with false discovery rate (FDR)‐adjusted significance values for each cell type. Dashed lines represent statistical significance threshold FDR < 0.1. (C) Prostate‐specific decomposition analysis using MuSiC on a single‐cell dataset from eight primary prostate specimens. The boxplot shows the estimated proportion of each cell‐type across the single‐cell clusters. (D) Uniform manifold approximation and projection (UMAP) projection of a single‐cell RNA‐Seq database comprising eight primary prostate specimens. The plot shows the expression level of the differentially expressed genes in the green module. Boxplots indicate the median (center line), interquartile range (box), and whiskers extending to the most extreme data points within the interquartile range. Two‐tailed Wilcoxon's tests were used to calculate *P*‐values.

Given the functional link between ECM processes and the stromal compartment, we hypothesized that PTEN protein loss could have an impact on the relative abundance of the diverse cell types in the prostate tumor. To explore compositional differences, we applied ESTIMATE, which uses predefined stromal and immune‐cell signatures to calculate stromal and immune scores and infer tumor purity (namely, the relative abundance of the epithelial compartment) from bulk transcriptomic data [30]. Tumor purity estimates were significantly decreased in the samples with PTEN protein loss, consistent with an increase in immune and stromal infiltration scores (Fig. 3A and Table S8). More detailed estimates for the abundances of 64 immune and stromal cell types using xCell [31] showed a notable increase in the relative abundance of fibroblasts in tumors exhibiting PTEN protein loss (Fig. 3B and Tables S9, S10). The deconvolution inference and ECM‐related functions jointly suggest that the transcriptomic landscape of PTEN protein‐deficient tumors involves compositional alterations in the tumor microenvironment.

To refine these findings, we next leveraged publicly available single‐cell RNA‐Seq data from prostate primary tumors [33] to perform cell‐type deconvolution (Fig. S3). Unlike xCell, which relies on bulk transcriptomic data and predefined marker genes, MuSiC incorporates single‐cell expression profiles to improve cell‐type resolution [32]. Consistent with our previous results, we observed a decrease in the luminal compartment and an increase in the endothelial and myofibroblast compartments in PTEN protein‐deficient tumors (Fig. 3C). Next, we analyzed the expression breadth of the genes impacted by PTEN protein loss with single‐cell resolution. Cells with the highest expression levels for the DEGs in the green module were confined to the nonimmune stromal compartment (Fig. 3D). Altogether, cell‐deconvolution and single‐cell resolution analyses support that PTEN protein loss in the prostate epithelium plays a major role in driving a dynamic response of the nonimmune tumor microenvironment, influencing the abundance of cancer‐associated fibroblasts and the tumor vasculature.

We next asked whether other early oncogenic events contribute to the stromal changes we associate with PTEN loss. ERG gene fusions, among the earliest genomic alterations in prostate cancer that precede *PTEN* deletion [16, 43], have been linked to altered stromal architecture and immune‐cell recruitment [44]. We applied STAR‐Fusion [36] to our RNA‐Seq data and identified TMPRSS2‐ERG in 53/197 (26.9%) tumors (Fig. S4, Table S11). While this frequency is lower than typically observed in fresh‐frozen cohorts [45, 46], it is consistent with expectations for FFPE‐derived RNA, where degradation limits fusion detection sensitivity [47]. Interestingly, fusion prevalence did not differ by PTEN IHC status (PTEN presence vs. PTEN loss; *P* = 0.456; Fig. S4). While TMPRSS2‐ERG positive cases showed higher tumor purity and reduced ESTIMATE stromal and immune scores, PTEN‐loss tumors maintained elevated stromal infiltration regardless of fusion status (Fig. S4). Similarly, the green module signature remained strongly associated with PTEN loss in both ERG fusion‐positive and ERG fusion‐negative primary tumors (Fig. S4). These results indicate that stromal remodeling linked to PTEN protein alterations occurs independently of the TMPRSS2‐ERG fusion.

### 3.4 PTEN loss in mouse models recapitulates ECM changes and stromal remodeling

**Fig. 4:**
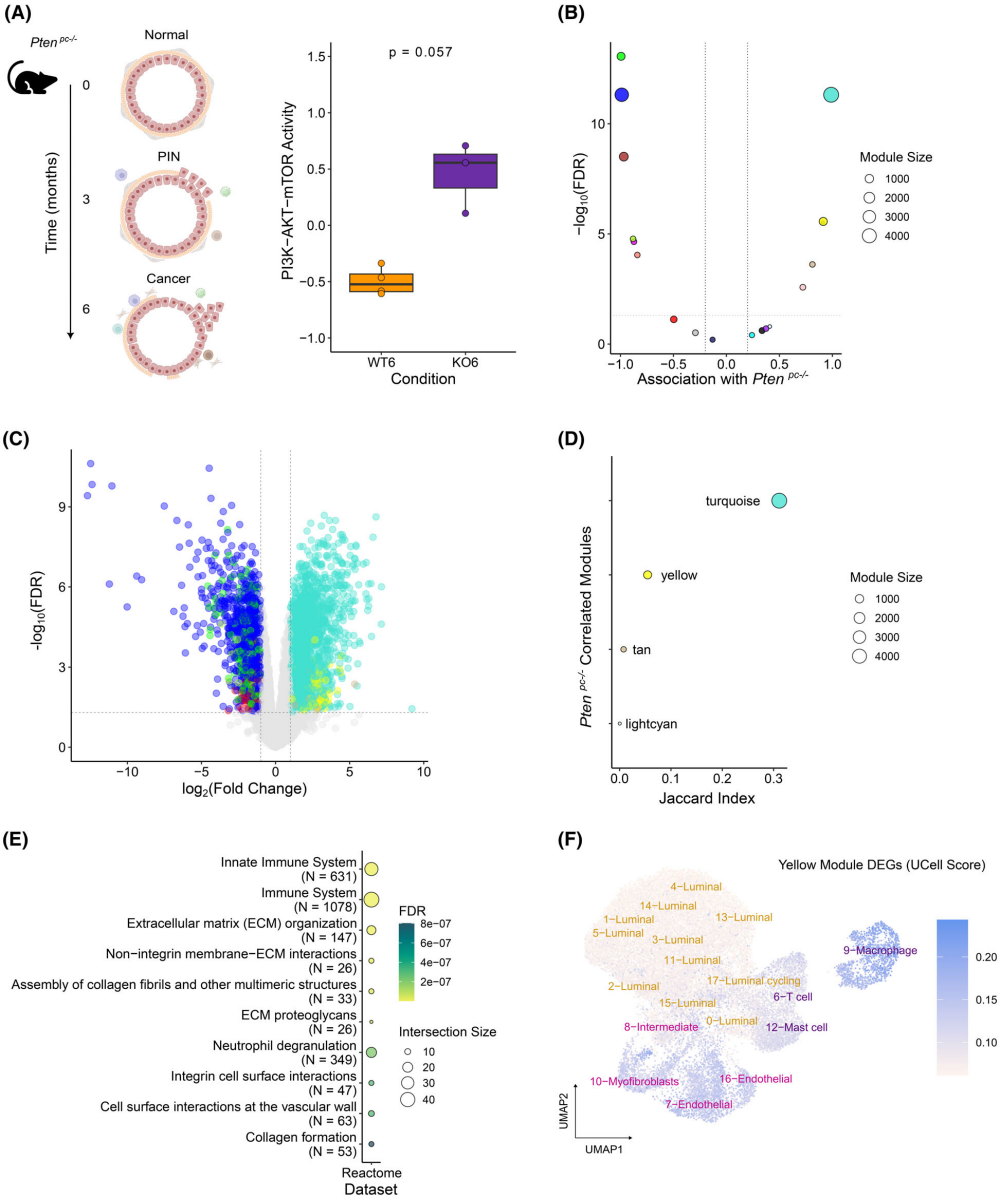
Stromal remodeling following *Pten* loss in mouse prostate cancer models. (A) Left panel: Illustration of the progression of the prostate‐specific, Cre‐LoxP *Pten* conditional knockout mouse model, showing progression to adenocarcinoma by 6 months. PIN: prostatic intraepithelial neoplasia. Right panel: *Prostate tissues from Pten conditional knockout* mice show increased PI3K‐AKT–mTOR signaling in the prostate at 6 months (WT: wild‐type vs. KO: knockout). Boxplots indicate the median (center line), interquartile range (box), and whiskers extending to the most extreme data points within the interquartile range. Two‐tailed Wilcoxon's test was used to calculate *P*‐value. (B) Volcano plot showing the results of the association (Pearson's *r*) of each Weighted Gene Correlation Network Analyses (WGCNA) with *Pten*‐null status. Colors indicate different gene co‐expression modules. Dot size represents the module size. Dashed lines represent statistical significance thresholds at |Pearson's *r*| > 0.2 and false discovery rate (FDR) < 0.05. (C) Volcano plot of differential gene expression analysis for *Pten* ^pc‐/‐^ vs *Pten*‐WT status. Each dot's color indicates the gene's WGCNA module membership. Dashed lines represent statistical significance thresholds |log_2_(fold change)| > 0 and FDR < 0.05. (D) Intersection between differentially expressed genes (DEGs) and WGCNA modules measured by the Jaccard Index. Colors indicate different gene co‐expression modules. Turquoise and yellow modules present the highest overlap with DEGs. (E) Reactome pathway enrichment analysis for DEGs in the yellow module, highlighting overrepresentation of immune and extracellular matrix (ECM)‐related pathways. (F) uniform manifold approximation and projection (UMAP) projection of single‐cell RNA‐Seq database from eight human primary prostate specimens. The plot shows the expression levels of DEGs in the yellow module.

Our analyses of human transcriptomes unveiled an association between PTEN loss and stromal remodeling. However, considering the complex genomic landscape of prostate adenocarcinoma [48] analysis of clinical specimens does not allow us to establish a direct causal relationship. To this end, we leveraged experimental murine models of prostate cancer with conditional *Pten* deletion specific to prostatic epithelial cells (by Cre recombinase under the control of the Probasin promoter, *Pten* ^*pc*−/−^). Generally, *Pten* ^*pc−*/−^ mice develop prostate intraepithelial lesions (PIN) by the age of 3 months, with cancer manifesting by 6 months (Fig. 4A left panel) [49]. As predicted, an increase of the PI3K‐AKT–mTOR signaling [42] was also evident in the transcriptomes of *Pten*‐deficient prostates at 6 months (Fig. 4A right panel).

We applied the module construction and differential gene expression workflow used in the human datasets (outlined in Fig. 2C) to murine prostate transcriptomic profiles from *Pten^pc‐/‐^*‐knockout and WT mice at 6 months. Two modules exhibited the strongest association with *Pten* loss in the murine model, which also presented the highest amount of DEGs (Fig. 4B–D, Fig. S5, Tables S12– S14). The first module, turquoise, was notably large, comprising 4680 genes and 1749 DEGs, and was primarily enriched in immune‐related pathways (Fig. S6). Interestingly, the DEGs in the second module, labeled yellow, exhibited significant enrichment in ECM‐associated mechanisms, such as ‘*Extracellular matrix organization’* (Fig. 4E, Table S15). Upon converting the DEGs in this module to their human homologs, we found that these were predominantly expressed in macrophages, endothelial cells and myofibroblasts in single‐cell data (Fig. 4F). Further deconvolution analysis using single‐cell RNA‐Seq revealed increased stromal cell contribution in prostate conditional *Pten*‐deficient mice (Fig. S7). These observations suggest that the absence of PTEN protein in the prostate epithelia induces critical changes in the stromal composition of tumors in both experimental models and clinical specimens.

### 3.5 PTEN protein loss in tumors is associated with activated TGF‐β signaling and senescence‐like secretory phenotype programs

To ascertain the molecular means behind the stromal remodeling induced upon tumor cell‐intrinsic PTEN protein loss, we focused on paracrine signals that could explain this phenomenon. Altered secretome profiles have been reported in *PTEN*‐deficient cells concomitant with the induction of cellular senescence [4]. Senescence is a combination of cell‐intrinsic arrest combined with a paracrine secretory program termed senescence‐associated secretory phenotype (SASP) [50, 51]. Notably, upon analysis of the differential transcriptome of PTEN‐deficient prostate cancer clinical specimens, we found a remarkable enrichment in the activated Transforming Growth Factor‐β (TGF‐β) pathway (Fig. 2F). TGF‐β is a key regulator of the senescence response, particularly in orchestrating stromal remodeling [50, 51, 52, 53]. Hence, we hypothesized that altered TGF‐β transcriptional programs and stromal remodeling were surrogate signals of the secretory reprogramming in PTEN‐deficient tumors, consistent with SASP induction.

**Fig. 5:**
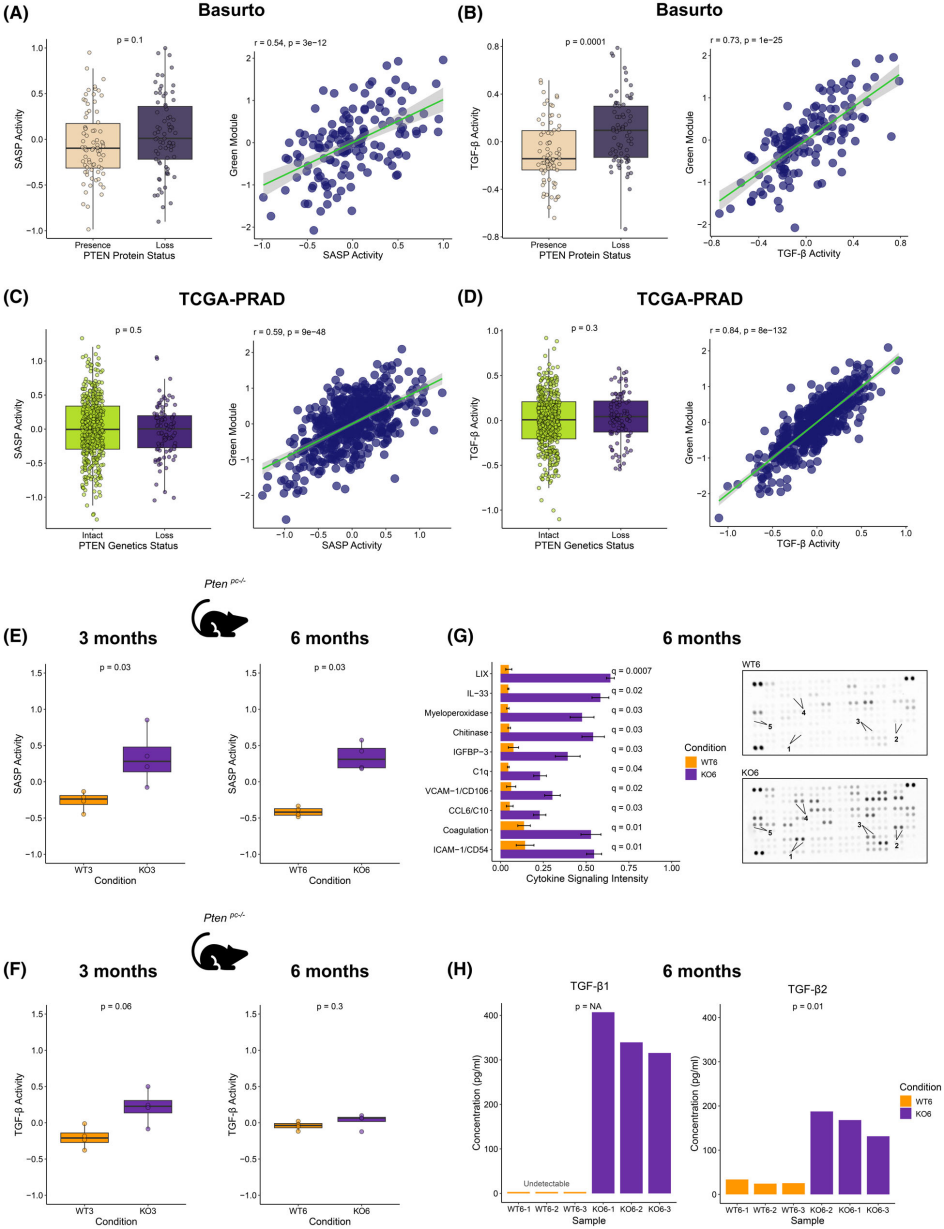
PTEN protein loss associates with senescence and TGF‐β. (A) Left panel: Senescence‐associated secretory phenotype (SASP) activity signature in our patient cohort according to PTEN protein loss and presence. Right panel: correlation between the SASP signature and the signature based on the DEGs in the green module in our cohort. (B) Left panel: TGF‐β signature (KEGG pathway in MSigDB) in our patient cohort according to PTEN protein loss and presence. Right panel: correlation between the TGF‐β signature and the signature based on the DEGs in the green module in our cohort. (C) Left panel: Senescence‐associated secretory phenotype (SASP) signature in the TCGA patient cohort according to PTEN gene copy loss. Right panel: correlation between the SASP signature and the signature based on the DEGs in the green module in the TCGA. (D) Left panel: TGF‐β signature in the TCGA‐PRAD patient cohort according to PTEN gene copy loss. Right panel: correlation between the TGF‐β signature and the signature based on the DEGs in the green module in the TCGA‐PRAD cohort. (E) SASP signature in the animal model with prostatic *Pten* deletion and wild‐type (WT) at three (left panel) and 6 months (right panel). (F) TGF‐β signature in the animal model with prostatic *Pten* deletion and WT at three (left panel) and 6 months (right panel). (G) Left panel: Cytokine signaling intensity for the top 10 most upregulated cytokines in 6‐month‐old prostate conditional *Pten*‐knockout (KO6) mice compared with age‐matched WT (WT6) controls according to fold change. Each cytokine was measured in duplicate (two technical replicates). Error bars indicate the standard error of the mean. Statistical significance was assessed using a two‐tailed unpaired *t*‐test; false discovery rate (FDR) *q*‐values are reported. Data were obtained from one experiment, including three biological replicates per group. Right panel: Two technical replicates for the top five most upregulated cytokines in KO6 and WT6 mice are highlighted and labeled on representative membranes: 1: LIX, 2: IL‐33, 3: Myeloperoxidase, 4: Chitinase, and 5: IGFBP‐3. (H). Relative abundance of TGF‐β1 (left panel) and TGF‐β2 (right panel) in 6‐month‐old *Pten*‐null mice compared with WT controls at the same age (KO6 vs WT6). TGF‐β1 levels were below detection in all WT samples. Two‐tailed *t*‐tests were used to calculate p‐values by comparing KO vs WT. Data were obtained from one experiment, including three biological replicates per group.

To this end, we explored the association of PTEN protein loss with senescence [54] and TGF‐β activation signatures ([55] and TGF‐β KEGG pathway in MSigDB) in different preclinical and clinical transcriptomics datasets. In our patient cohort, we consistently observed an upregulation of these pathways in the primary tumors exhibiting PTEN protein loss and a strong correlation with the signature based on the DEGs in the green module (hereafter referred to as the ‘green module signature’, comprised of 75 genes, Table S6). These associations are illustrated in Fig. 5A,B, and Fig. S8. Interestingly, the associations of stromal remodeling with SASP and TGF‐β signaling were validated in the TCGA‐PRAD cohort, yet the association with genetic loss was not significant (Fig. 5C,D), suggesting *PTEN* genetic calls alone fail to capture this functional program. Consistently, we also observed these trends in our mouse models upon *Pten* deletion (Fig. 5E,F).

To experimentally validate the link between PTEN loss and a senescent‐associated secretory phenotype, we compared cytokine profiles on prostate conditional *Pten‐*null and *Pten‐*WT prostate specimens from our mouse model. Using a multiplex cytokine array, we observed a significant upregulation of cytokines in *Pten*‐deficient prostates, with 32 out of 111 analytes showing FDR < 0.05 (two‐sided *t*‐test; Table S16). Among the top 10 significantly upregulated cytokines (Fig. 5G), we identified key mediators of senescence‐associated inflammation and tissue remodeling. These include canonical SASP factors and stromal remodeling markers, such as IL‐33, IGFBP‐3, VCAM‐1, and ICAM‐1, which have been previously associated with cellular senescence and tumor microenvironment remodeling in various cancer contexts [50, 51, 56]. Their upregulation in *Pten*‐deficient prostates supports the activation of a paracrine senescence program involving both epithelial and stromal compartments. The observed combination of secreted inflammatory mediators and stromal response factors is consistent with a model in which epithelial *Pten* loss induces a SASP that drives stromal reprogramming.

To further validate these findings, we quantified active cytokine levels by ELISA in the same mice and confirmed a significant increase in both TGF‐β1 and TGF‐β2 in *Pten*‐null tissues compared with *Pten*‐WT controls (Fig. 5H and Table S16). Together, these complementary assays substantiate our transcriptomic findings, demonstrating that epithelial PTEN loss triggers TGF‐β production and a senescence‐associated secretory program consistent with ECM remodeling.

### 3.6 A green module‐based signature sub‐stratifies PTEN‐deficient prostate cancers

**Fig. 6:**
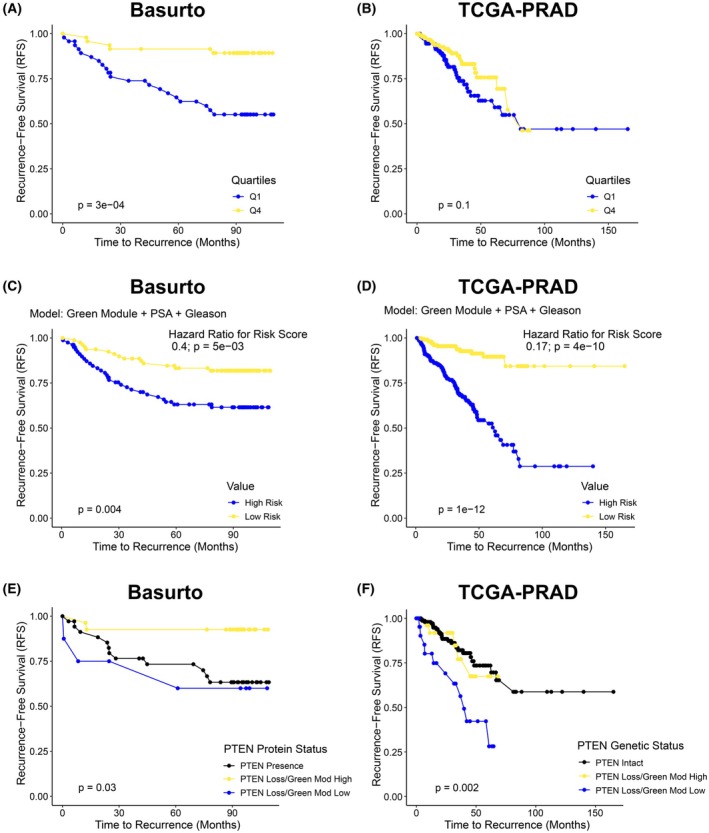
Senescence‐associated green module signature improves risk stratification. (A) Recurrence‐free survival in Basurto cohort stratified into low (Q1) and high (Q4) expression of green module signature (Q1 *n* = 47 with 20 events, Q4 *n* = 47 with 5 events); log‐rank *P* reported. (B) Recurrence‐free survival in TCGA‐PRAD cohort stratified into low (Q1) and high (Q4) expression of green module signature (Q1 *n* = 123 with 30 events, Q4 *n* = 123 with 21 events); log‐rank p reported. (C) Recurrence‐free survival in the Basurto cohort, stratified into high‐risk and low‐risk groups based on the median of a combined prognostic score derived from a multivariate Cox model, including the green module signature, PSA, and Gleason score; log‐rank *P* reported. (D) Independent validation in the TCGA‐PRAD cohort using the same multivariate Cox model. Patients were stratified into high‐risk and low‐risk groups according to the median of a combined prognostic score; log‐rank *P* reported. (E) Recurrence‐free survival in Basurto cohort with immunohistochemistry PTEN loss, stratified by high versus low green module signature (Q1 vs. Q4; Q1 *n* = 8 with 3 events, Q4 *n* = 27 with 2 events); log‐rank *P* reported. (F) Recurrence‐free survival in TCGA‐PRAD cases with bi‐allelic *PTEN* loss, stratified by high versus low green module signature (Q1 vs. Q4; Q1 *n* = 46 with 16 events, Q4 *n* = 48 with 6 events); log‐rank *P* reported.

PTEN loss elicits a tumor‐suppressive senescence response that is evaded in the course of tumor progression [49, 52, 57]. In turn, the clinical assessment of PTEN loss might introduce an important confounding factor, as it would not differentiate between PTEN‐deficient tumors with senescence (indolent) and PTEN‐deficient tumors that have evaded senescence (aggressive). We therefore hypothesized that computing the senescence paracrine tumor signaling via our green module signature would add significant clinical information to the assessment of PTEN loss. We first asked whether the green module signature alone could stratify recurrence‐free survival. In our Basurto University Hospital cohort (total *n* = 188; 49 events), patients with high expression levels (quartile 4, Q4) of the green module signature associated with markedly better recurrence‐free survival than those in the bottom quartile Q1 (Fig. 6A, log‐rank *P* = 0.0003). These results are aligned with the association of this module to the tumor‐suppressive senescence paracrine program. The same quartile stratification in the TCGA‐PRAD cohort (*n* = 491; 91 events) corroborated the protective trend of this gene set (Fig. 6B, log‐rank *P* = 0.0997).

To test the clinical utility of the green module signature, we built multivariable Cox models incorporating PSA and Gleason grade, with or without the green module signature. In the Basurto cohort, adding the green module significantly improved model performance (likelihood ratio, LR, *χ* ^2^ = 28.8 on 3 df vs. 20.7 on 2 df; *P* = 2.5 × 10^−6^ vs. 3.2 × 10^−5^, Table S17), and yielded robust separation of high‐ versus low‐risk groups (Fig. 6C, log‐rank *P* = 0.004). Each standard deviation increase in the z‐scored green module signature corresponded to a 32% reduction in recurrence risk (HR = 0.68; 95% CI 0.52–0.89, *P* = 0.004, Table S17). In TCGA‐PRAD, adding the green module similarly enhanced fit (LR, *χ* ^2^ = 52.4 to 56.1; both *P* < 10^−14^) with a clear separation of risk‐score strata (Fig. 6D, log‐rank *P* = 1.4 × 10^−12^) and protective trend (HR = 0.82; 95% CI 0.65–1.03, *P* = 0.07, Table S17).

Finally, we asked whether the green module could sub‐stratify PTEN‐deficient tumors. In the Basurto cohort, PTEN protein‐deficient cases exhibited a trend toward better prognosis (Fig. S9A, log‐rank *P* = 0.1). Importantly, Kaplan–Meier curves comparing extreme green module quartiles (Q4 vs. Q1) revealed a group of patients with PTEN loss and high green module (reminiscent of PTEN‐negative tumors with an active senescence process) that were responsible for the better prognosis ascribed to PTEN protein deficiency (Fig. 6E, long‐rank *P* = 0.002). Similarly, the TCGA prostate cancer subset with bi‐allelic *PTEN* deep deletion (*n* = 368; 69 events) exhibited a consistent prognostic difference when the activity of the green module was computed (Fig. 6F, long‐rank *P* = 0.002), compared with the modest prognostic capacity of PTEN genetic status alone (Fig. S9, long‐rank *P* = 0.1).

For context, we compared the performance of the green module with the PI3K‐AKT–mTOR signature previously used in our analyses [42]. Unlike the green module, the PI3K signature did not significantly stratify recurrence‐free survival in either cohort (Fig. S10). In multivariable Cox models adjusted for PSA and Gleason, the PI3K‐AKT–mTOR signature showed negative coefficients and resulted in smaller improvements in model performance (Basurto LR, *χ* ^2^ = 28.8 vs. 20.8; TCGA 56.1 vs. 53.23), as well as lower concordance indices (Basurto C‐index = 0.697 vs 0.624; TCGA: 0.731 vs. 0.709, Table S17). By contrast, the green module provided consistent and stronger prognostic information in both cohorts. These observations support the notion that the stromal/senescence axis captured by the green module reflects a distinct dimension of PTEN biology not encompassed by tumor‐intrinsic PI3K‐related activity.

Together, these results demonstrate that the green module signature (i) predicts recurrence on its own, (ii) enhances risk models based on PSA and Gleason grade, and (iii) identifies PTEN‐deficient tumors that retain a tumor‐suppressive senescence‐associated stromal response.

## 4 Discussion

In this study, we adopted a data‐driven discovery framework aimed at defining stromal biomarkers of PTEN loss with clinical utility. Genomic loss of *PTEN* represents one of the most robustly reproducible and extensively validated genetic alterations in prostate cancer. However, DNA assays alone miss non‐genomic inactivation mechanisms, and IHC, while spatially precise, does not report on downstream molecular consequences. In this study, we assembled a novel prostate cancer clinical cohort (*n* = 197, from Basurto University Hospital in Spain) and quantified the loss of PTEN protein under stringent controls, including a clinical grade IHC protocol, paired with RNA‐Seq to perform a potent computational integrative study. To account for *intratumoral heterogeneity*, reported in ~50% of cases [16, 58, 59], we sampled two tumor cores per patient. To establish a cutoff that would capture the loss of PTEN functionality, we integrated H‐scores with a surrogate of PI3K‐AKT–mTOR transcriptional activity.

Our analyses identified PTEN protein loss in about 50% of the patients in our cohort, which is higher than the loss reported at the genomic level in primary tumors (15–20%) [1, 2, 3] while in line with previous reports. For example, Lotan et al. [60] reported that only 66% of primary tumors with PTEN protein loss presented *PTEN* genomic deletion by fluorescence *in situ* hybridization. This discrepancy supports the need for tissue‐based protein assays to more accurately capture the complexity of PTEN activity loss within prostate primary tumors.

We further capitalized on our RNA‐Seq data to identify transcriptome‐wide changes associated with PTEN protein loss. By combining pathway‐based and agnostic gene‐network approaches, we identified a group of co‐expressed genes related to ECM functions as the main cancer‐cell‐extrinsic process differentially associated with PTEN protein loss. *In silico* cell‐type deconvolution analyses and integration with transcriptomic datasets at single‐cell resolution revealed an expansion of the stromal compartment in association with PTEN protein loss and a concomitant upregulation of ECM‐associated genes that were confined to the nonimmune stroma.

Prostate primary tumors harbor numerous genomic, transcriptomic, and proteomic alterations, with PTEN loss co‐occurring alongside these changes. This complexity highlights the correlative nature of clinical studies, which we mitigated through the implementation of animal models with tissue‐specific deletion of *Pten*, thus allowing us to extract those molecular processes in human prostate cancer that are causally associated with PTEN loss. By employing genetically modified prostate‐specific *Pten*‐knockout mice, we observed a tumoral transcriptomic rewiring and stromal reprogramming that matched our observations in human specimens. Together, these data suggest a causal link between PTEN loss and stromal reaction in prostate cancer. These patterns agree with the phenomenon of stromal reaction described in several solid tumors, including the prostate, linked to tumor growth, invasion, and metastasis [61, 62]. Reactive stroma in prostate cancer involves a transition of cancer‐associated fibroblasts (CAFs) into myofibroblasts and inflammatory CAFs, remodeled ECM, activated angiogenic niche, and an immunosuppressive landscape [62, 63].

Single‐cell resolution analyses suggest that the green module signature is predominantly expressed in stromal populations, as opposed to conventional SASP signatures. The green module may capture a coordinated senescence program initiated by epithelial PTEN loss, encompassing both the epithelial production of SASP factors and the transcriptional response of the surrounding stroma to these paracrine signals. Thus, rather than marking a purely stromal process, the green module captures the broader paracrine senescence axis. In this context, the signature may serve as a proxy for a tumor‐suppressive program [52] involving both epithelial and stromal compartments, which could explain its association with reduced risk of recurrence.

Importantly, the use of this signature could allow a better stratification of PTEN‐deficient prostate tumors. We find that PTEN protein loss is associated with a trend toward better prognosis, which is counterintuitive with the tumor‐suppressive activity of the phosphatase. Importantly, when we account for the senescence‐associated stroma remodeling signature contained in the green module, we are able to differentiate between two types of PTEN‐deficient tumors: those that retain a high signature (which we associate with tumors with an active senescence response, and therefore indolent) and those with lower signature activity (which we postulate as tumors that have evaded the senescence response elicited by PTEN loss, and therefore more aggressive). This green module‐based stratification is also valuable when assessing PTEN genetic loss, thus suggesting that it might provide an important layer of molecular information for clinical assessment.

While our bulk RNA‐Seq analyses effectively uncovered stromal remodeling programs, they lack the single‐cell or spatial resolution needed to precisely define the paracrine interactions between epithelial and stromal compartments. Moreover, our human cohort data are inherently correlative; although the *Pten*‐knockout mouse experiments support causality, definitive proof will require targeted *in vivo* perturbations of the senescence‐SASP axis in prostate tumors. Future studies using single‐cell or spatial transcriptomics will be essential to resolve cell‐type specific contributions and validate these interactions directly. In addition, although we assessed genomic confounding through gene fusion status, other alterations such as *TP53*, *SPOP*, or *MYC* could also influence stromal responses to PTEN loss. While these events are less frequent, future large‐scale genomic studies will be important to formally rule out their contribution. Together, these future directions will be essential for translating our findings into therapeutic strategies.

## 5 Conclusion

This study leverages a publicly accessible, novel patient cohort with integrated PTEN IHC and RNA‐Seq to map the downstream effects of PTEN protein loss in prostate cancer. We demonstrate that PTEN deficiency activates a senescence‐associated stroma remodeling program in both human tumors and *Pten*‐knockout mice. Implementing such a transcriptional signature could help refine PTEN‐based patient stratification to guide clinical decisions.

## Conflict of interest

The authors declare no conflict of interest.

## Author contributions

IR‐L and SG‐L performed bioinformatic analyses on bulk and single‐cell transcriptomic datasets and contributed to the preparation of the figures for the manuscript. IM supervised the work of IR‐L and SG‐L. MU and AL‐I generated the Basurto cohort and provided biological specimens supported by SR and AS‐M, IA and AZ coordinated the experimental preparation of the IHC under the supervision of AC. MZ and AU‐O selected the areas for the TMAs. IHC experiments were performed by PN. JIL performed the IHC quantification. LB‐B and NM‐M performed the cytokine array and ELISA experiments and quantifications. MG contributed to the study design. AC and IM conceived the study, supervised the execution of the project, and wrote the manuscript. All authors have read and approved the final version of the manuscript.

## Supporting information


**Fig. S1.** PI3K pathway activity and PTEN mRNA expression by PTEN protein status.

**Fig. S2.** Gene network analyses using WGCNA in the human dataset.

**Fig. S3.** Functional enrichment and expression patterns of the purple module.

**Fig. S4.** Association of ERG gene fusions with stromal infiltration and extracellular matrix (ECM) signature.

**Fig. S5.** Gene network analyses using WGCNA in the mouse dataset.

**Fig. S6.** Functional enrichment analyses of the differentially expressed genes in the turquoise module.

**Fig. S7.** Deconvolution analysis of the mouse transcriptomic data using MuSiC.

**Fig. S8.** Loss of PTEN protein increases the transcriptional activity of the TGF‐β response signature (TBRS) across different cell types.

**Fig. S9.** Recurrence‐free survival analyses with PTEN status.

**Fig. S10.** Recurrence‐free survival analyses with PI3K–AKT–mTOR signature.

**Table S1.** Patient characteristics of the Basurto cohort. The table includes age, DV200, PSA, disease‐free survival status, disease‐free survival time (in months), Gleason grading pattern (primary and secondary grades reported by the pathologist) Gleason grading score (adding the primary and secondary Gleason patterns), H‐scores and PTEN protein status.

**Table S2.** Immunohistochemistry quality assessment results. The column *stroma negative* indicates the number of tissue cores from that case showing negative PTEN staining in the stromal compartment, while stroma positive indicates the number of cores with positive stromal PTEN staining. The *Core absent* column captures the number of tissue cores that were missing or could not be evaluated due to absence on the slide. The *No tumor* column denotes the number of cores that did not contain tumor tissue. The Staining failure column reflects cases where stromal PTEN staining could not be assessed due to technical issues or ambiguous staining patterns. The *Included or Excluded* column specifies whether the case was ultimately included in the final analysis. The *Comments* field contains relevant observations.

**Table S3.** Gene Set Enrichment Analyses (GSEA) results for the human transcriptome comparing PTEN‐loss and PTEN‐presence. Each row represents a significantly enriched hallmark gene set. The *NAME* column indicates the biological process or pathway. *GS.br..follow.link.to.MSigDB* corresponds to the MSigDB gene set name. *SIZE* refers to the number of genes in the gene set used in the analysis. *ES* (Enrichment Score) reflects the degree to which the gene set is overrepresented at the extremes (top or bottom) of the ranked list. *NES* (Normalized Enrichment Score) accounts for differences in gene set size and correlations between gene sets and the dataset. *NOM.p.val* is the nominal *P*‐value, *FDR.q.val* is the false discovery rate q‐value, and *FWER.p.val* is the family‐wise error rate *P*‐value. *RANK.AT.MAX* indicates the position in the ranked gene list where the enrichment score reaches its maximum. *LEADING.EDGE* shows a summary of the subset of genes contributing most to the enrichment signal (including the percentage of genes in the core enrichment subset (tags, their position in the ranked list), and their enrichment contribution, signal).

**Table S4.** Results of the Weighted Gene Co‐expression Network Analyses (WGCNA) in the patient cohort. Each row corresponds to a gene. Columns prefixed with *MM* represent module membership values or the gene in each WGCNA module, reflecting its correlation with the module eigengene. *moduleColors* indicate the module to which the gene was ultimately assigned based on maximum membership. The *GS*. columns represent gene significance values for various traits (e.g., *GS.Age*, *GS.PTEN*_*Exp_log2*, *GS.DFS.STATUS*), quantifying the correlation between gene expression and each phenotype. Network connectivity metrics include *kTotal* (overall connectivity), *kWithin* (connectivity within the assigned module), *kOut* (connectivity outside the module), and *kDiff* (difference between within‐module and outside‐module connectivity).

**Table S5.** Correlation between WGCNA module eigengenes and clinical or molecular phenotypes. Each row represents a module eigengene (*ME_name*), which is the first principal component summarizing the expression pattern of genes in that module. Columns include Pearson correlation coefficients (*Pearson_Cor) between each module and the indicated phenotypic variables (e.g., Age, DV200, DFS.TIME, PTEN_status, etc.), as well as their corresponding multiple testing‐adjusted FDR values (*FDR).

**Table S6.** Results of differential expression analyses of the patient cohort using the limma‐voom pipeline. Sheet1: The table includes log_2_ fold changes (*log2FC_limma_voom*), average expression (*AveExpr*), moderated *t*‐statistics (*t*), raw and adjusted *P*‐values (*pvalues_limma_voom*, *padj_limma_voom*), *B*‐statistics (*B*), and gene identifiers (*GeneID, gene_name*). Sheet 2: List of 75 genes that comprise the green module signature, obtained from intersecting the green WGCNA module with the differentially expressed genes.

**Table S7.** Results of functional enrichment for the differentially expressed genes in the green module by gprofiler. Each row corresponds to one enriched *term. term_id*, *term_name*, and *source* identify the enriched term and its annotation source (e.g., GO, KEGG). The *P_value* column indicates the statistical significance of overrepresentation, and *intersection_size* reports the number of query genes overlapping with the term. *term_size* is the number of genes annotated to the term in the background database, and *query_size* is the total number of input genes. precision and recall describe the proportion of intersecting genes relative to the query and term size, respectively.

**Table S8.** Results of *in silico* deconvolution by ESTIMATE. The table reports stromal and immune cell infiltration scores, as well as inferred tumor purity for each sample. The *stromal* and *immune* columns represent the stromal and immune scores, respectively, based on expression of signature gene sets. The *estimate* column reflects the overall level of nontumor content (immune + stromal). Tumor purity (*purity* column) was inferred from the ESTIMATE score using the algorithm's standard transformation.

**Table S9.** Results of xCell *in silico* deconvolution of the human transcriptome dataset. The table lists the estimated relative abundance scores for a wide range of immune and stromal cell populations (columns) inferred by xCell for each sample ID (row).

**Table S10.** Calculation of differential estimates by xCell *in silico* deconvolution for PTEN loss and presence categories in Basurto cohort. The table shows the results of statistical comparisons for each cell type, including the *P*‐value, false discovery rate (*FDR*), direction of the change (*Direction Mean value PTEN protein loss* – *presence* where Positive indicates higher enrichment in PTEN‐loss tumors; Negative indicates higher enrichment in PTEN‐intact tumors), and the magnitude of the change (*mean value PTEN‐protein loss* – *PTEN‐presence*).

**Table S11.** Gene fusions identified in our cohort using STAR‐Fusion and FusionInspector. The table includes the sample ID, fusion name, number of detected fusion events per sample, counts of junction reads and spanning fragments supporting the fusion, as well as the mean estimated junction (*Mean_est_J*) and spanning fragment (*Mean_est_S*) support. *LeftGene* and *RightGene* denote the fused genes, annotated with their Ensembl gene identifiers. The column *MultipleEvents* indicates whether multiple fusion variants were detected for a given gene pair in the same sample.

**Table S12.** Results of the Weighted Gene Co‐expression Network Analyses (WGCNA) in the mouse transcriptomes. Each row corresponds to a gene (*Gene ID*). Columns prefixed with *MM* represent module membership values for the gene in each WGCNA module, reflecting its correlation with the module eigengene. *moduleColors* indicates the module to which the gene was ultimately assigned based on maximum membership. The *GS*. columns represent gene significance values for various phenotypes (e.g., KO vs WT), quantifying the correlation between gene expression and each phenotype. Network connectivity metrics include *kTotal* (overall connectivity), *kWithin* (connectivity within the assigned module), *kOut* (connectivity outside the module), and *kDiff* (difference between within‐module and outside‐module connectivity).

**Table S13.** Association between WGCNA module eigengenes and genotype (KO6 vs WT6) in mouse samples. The table lists the Pearson correlation coefficient between each module eigengene (*Module_name*) and genotype (*KO6.vs.WT6_Association*), along with the associated false discovery rate (*KO6.vs.WT6_FDR*).

**Table S14.** Results of differential expression analyses of the mouse dataset using the limma‐voom pipeline. Sheet 1: The table includes log_2_ fold changes (*log2FC_limma_voom*), average expression (*AveExpr*), moderated *t*‐statistics (*t*), raw and adjusted *P*‐values (*pvalues_limma_voom*, *padj_limma_voom*), *B*‐statistics (*B*), and gene identifiers (*GeneID, gene_name*). Sheet 2: List of 185 genes comprising the yellow module signature, obtained by intersecting the yellow WGCNA module with the differentially expressed genes.

**Table S15.** Results of functional enrichment for the differentially expressed genes in the yellow mouse module by gprofiler. Each row corresponds to one enriched term. *term_id, term_name*, and source identify the enriched term and its annotation source (e.g., GO, KEGG). The *P_value* column indicates the statistical significance of overrepresentation, and *intersection_size* reports the number of query genes overlapping with the term. *term_size* is the number of genes annotated to the term in the background database, and *query_size* is the total number of input genes. precision and recall describe the proportion of intersecting genes relative to the query and term size, respectively.

**Table S16.** Results for the experimental validation of the cytokine array, TGF‐β1 and TGF‐β2 ELISA experiments. Sheet 1: cytokine signaling intensities for three PTEN‐null (HO) and three PTEN‐WT samples, including their means, standard deviations (sd), the fold change, *P*‐values and FDR‐adjusted *q*‐values for each cytokine. Sheets 2 and 3: ELISA quantifications of TGF‐β1 and TGF‐β2, respectively. Each sheet includes the condition (WT or HO), mouse IDs, TGF‐β concentration in pg·mL^−1^, and the fold change relative to the average of the WT group. In cases where ELISA values were negative, these likely represent measurements below the detection limit and are noted accordingly in the representations.

**Table S17.** Multivariable Cox proportional‐hazards analysis of recurrence‐free survival. Hazard ratios (HRs), 95% confidence intervals (CIs), and *P*‐values for serum PSA, and Gleason score, alone or in combination with the green module or PI3K‐AKT–mTOR signatures, fitted in the Basurto cohort (sheet 1) and the TCGA‐PRAD cohort (sheet 2).

## Data Availability

The raw data generated in this project are available in the European Nucleotide Archive (ENA) under project accession number: PRJEB49285. Processed data are available at GEO at accession number: GSE288484. The code for data analyses is available at https://github.com/imendizabalCIC/PTEN_protein_loss_project/.
