# Supplementary figures and images for: Transcriptional network analysis of PTEN‐protein‐deficient prostate tumors reveals robust stromal reprogramming and signs of senescent paracrine communication
Source: Mol Oncol. 2025 Nov 17;20(6):1429–52. doi: 10.1002/1878-0261.70164 (PMC13238722; doi:10.1002/1878-0261.70164)

**A**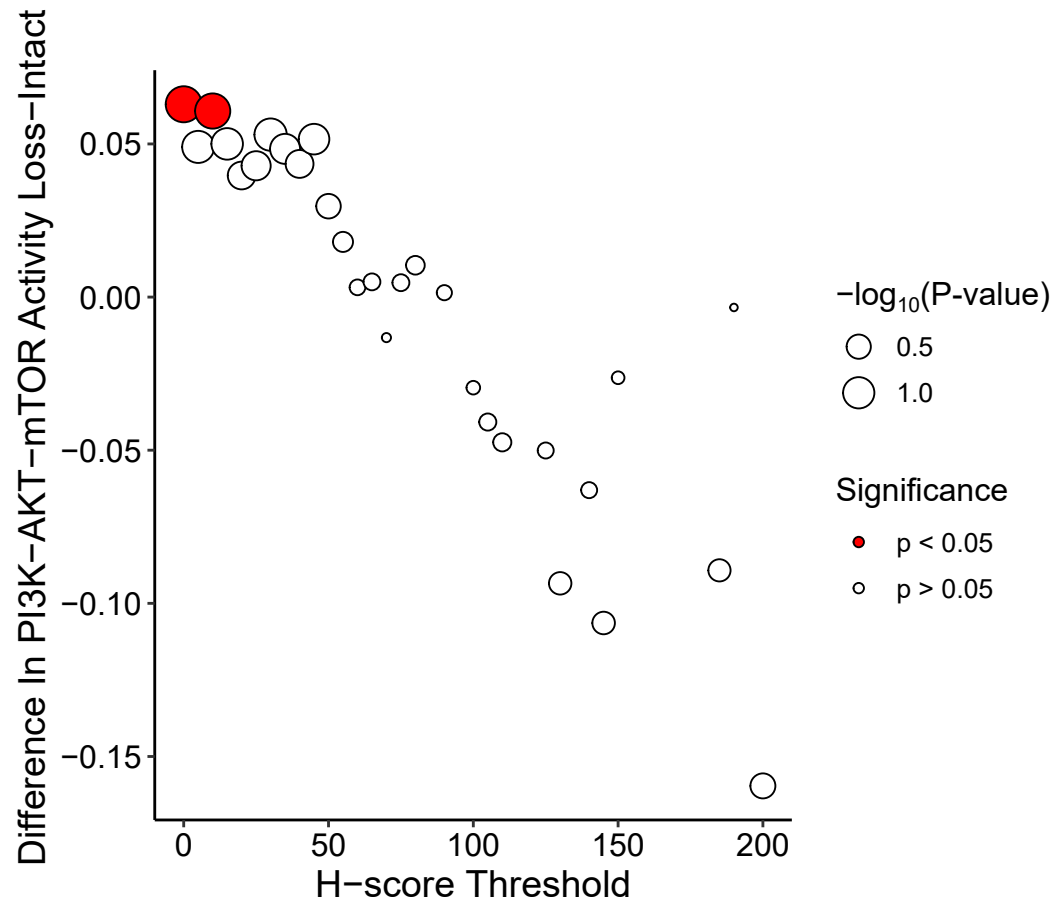**B**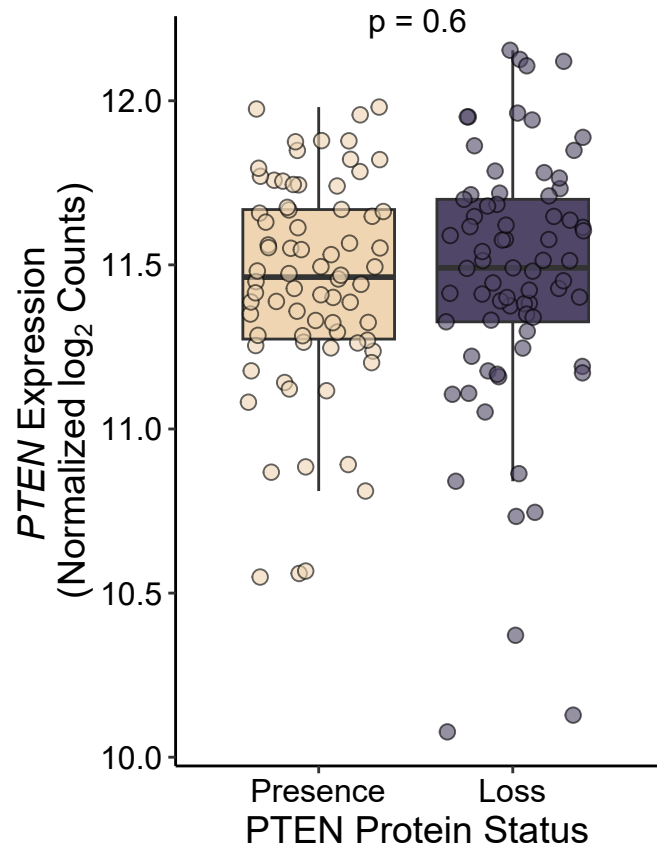

Supplement: Supplementary file 1 — Fig. S1. PI3K pathway activity and PTEN mRNA expression by PTEN protein status. [file MOL2-20-1429-s008.pdf]

A

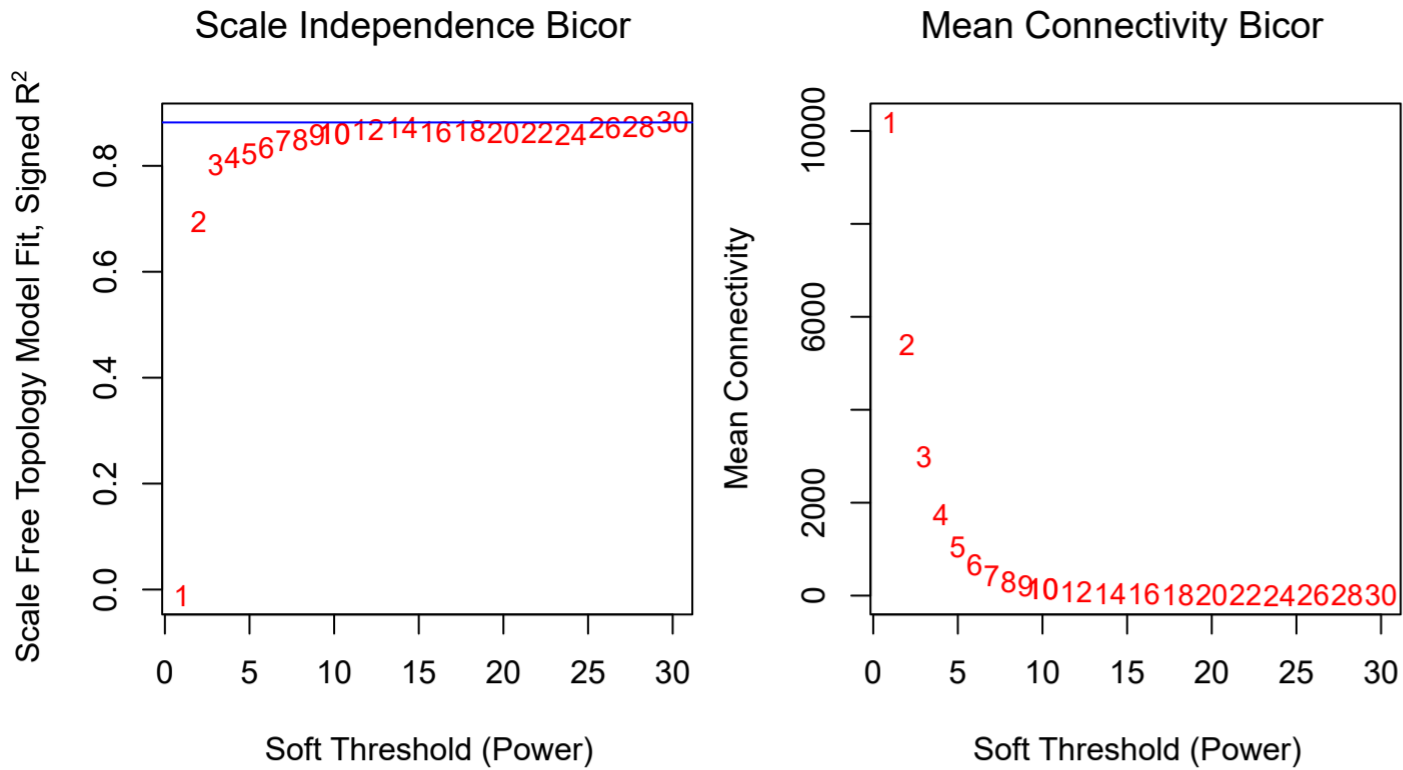

B

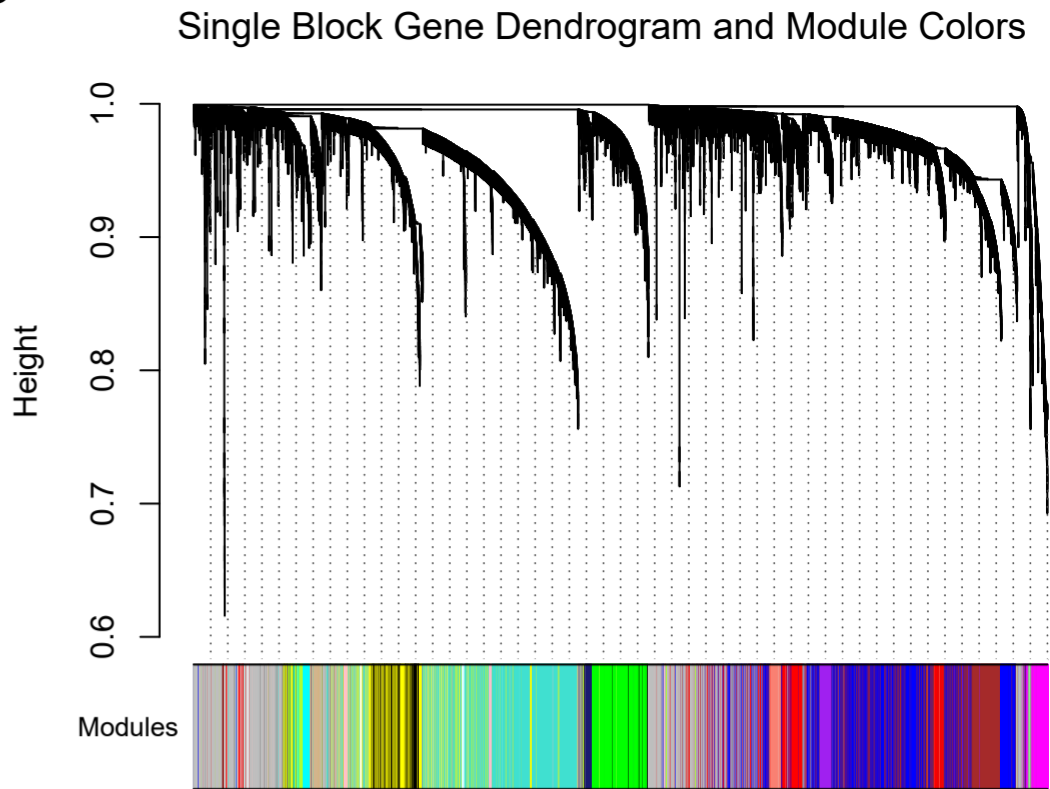

C

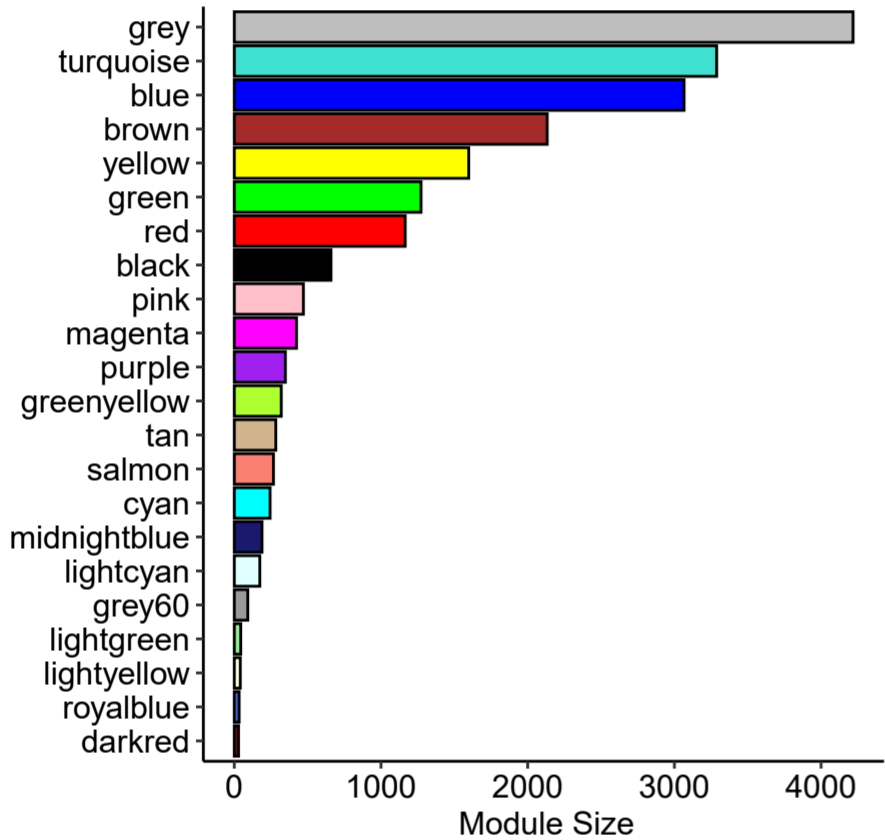

D

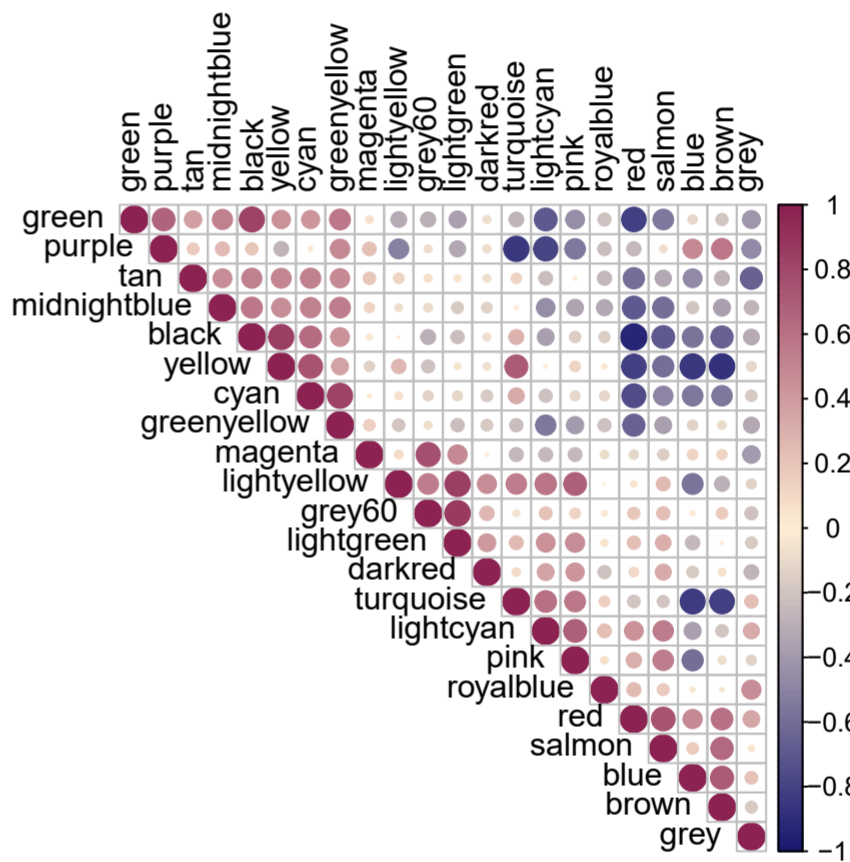

E

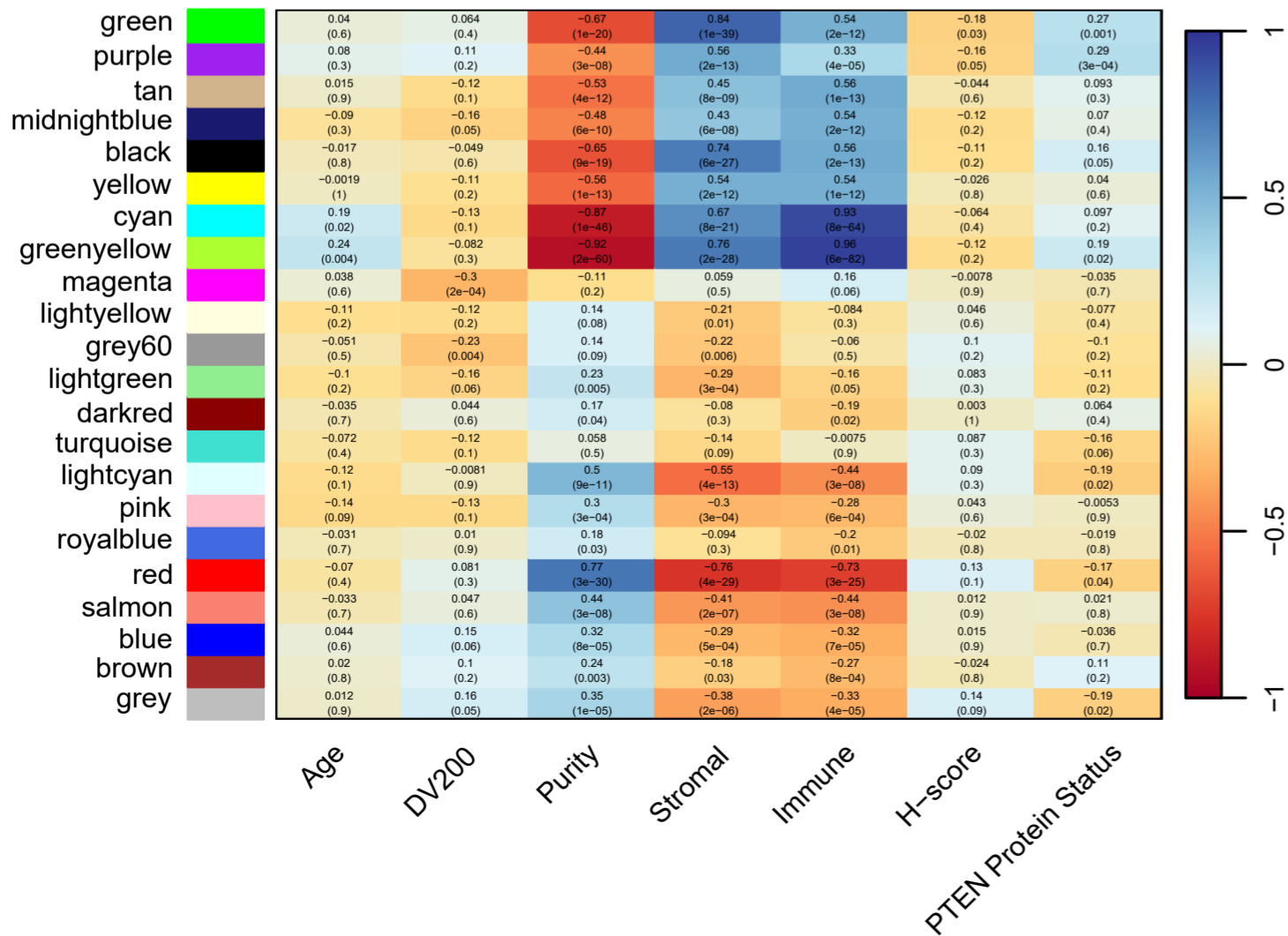

Supplement: Supplementary file 2 — Fig. S2. Gene network analyses using WGCNA in the human dataset. [file MOL2-20-1429-s002.pdf]

**A**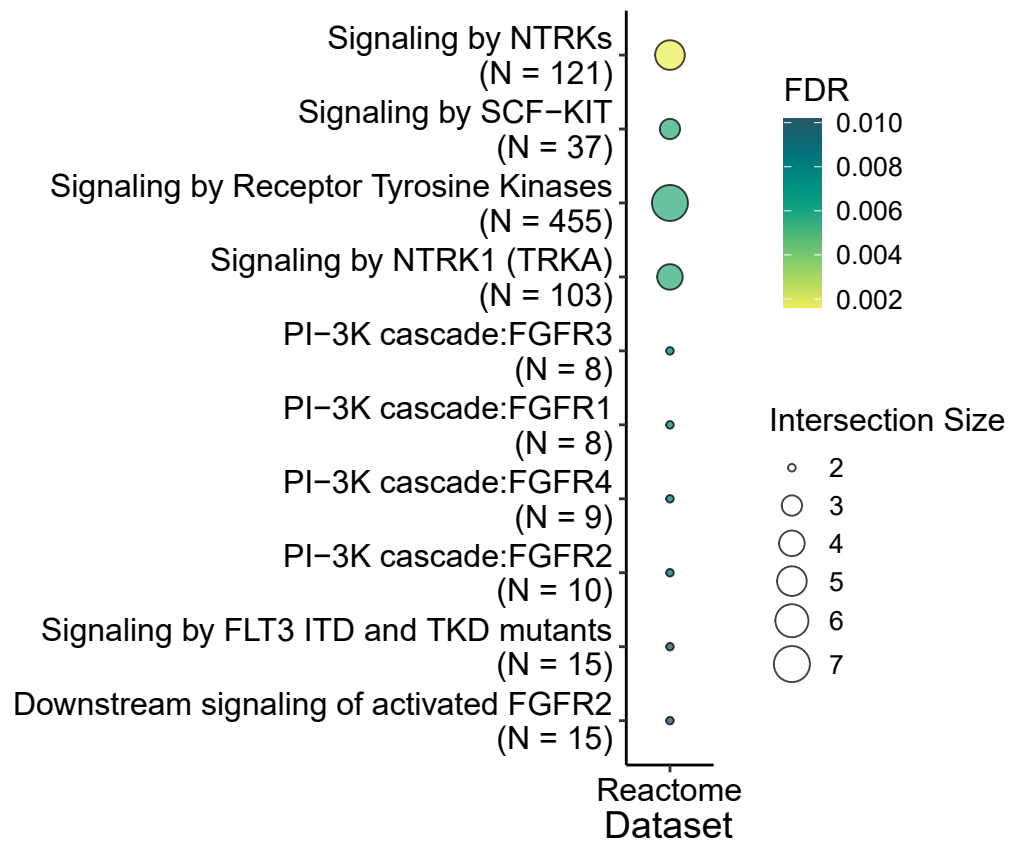**B**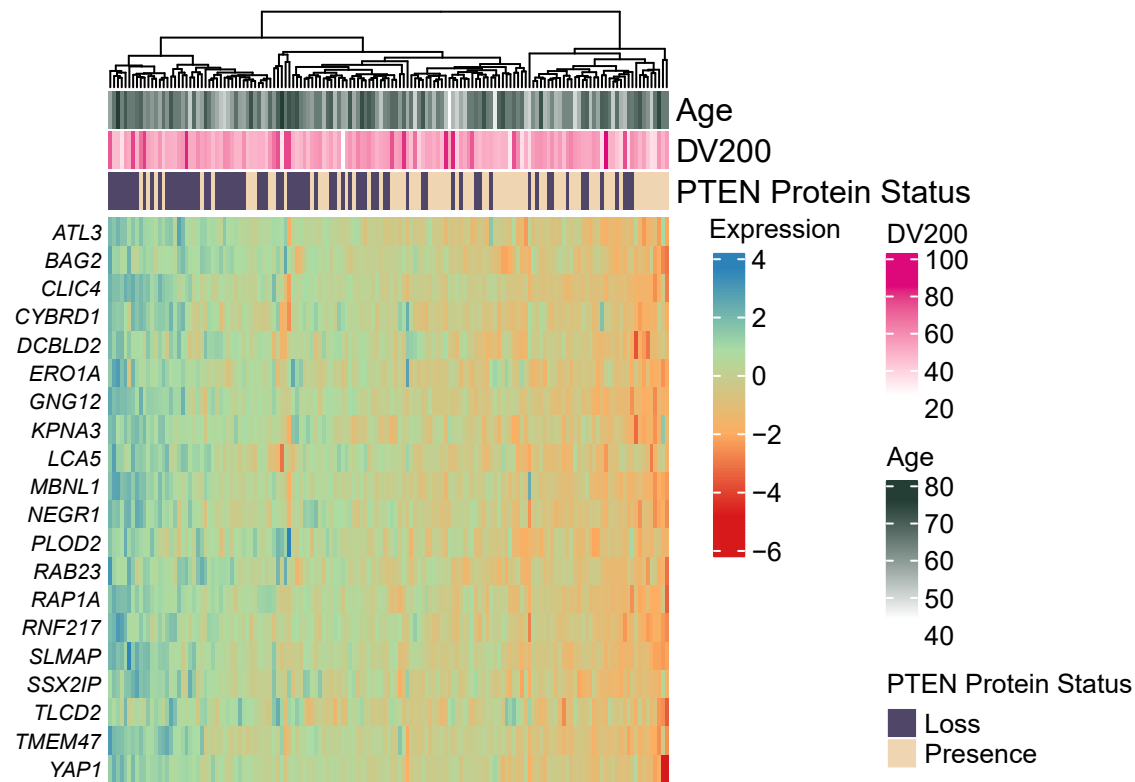**C**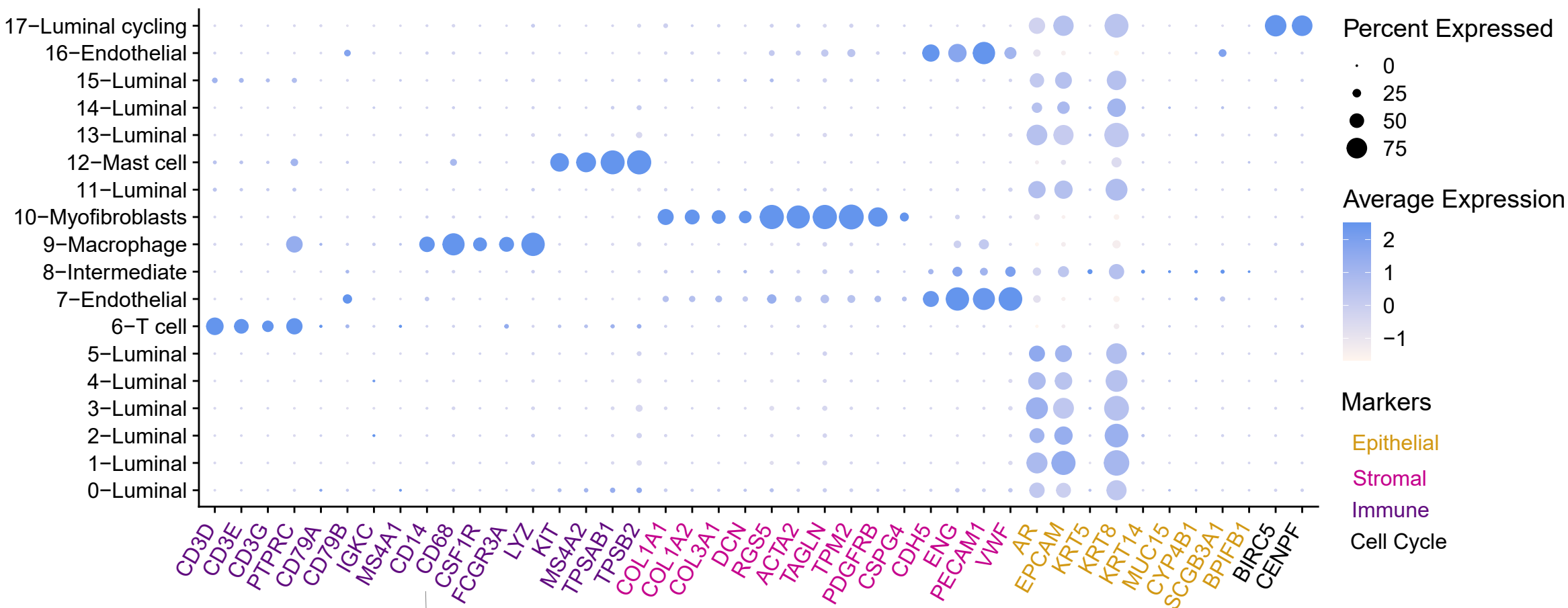**D**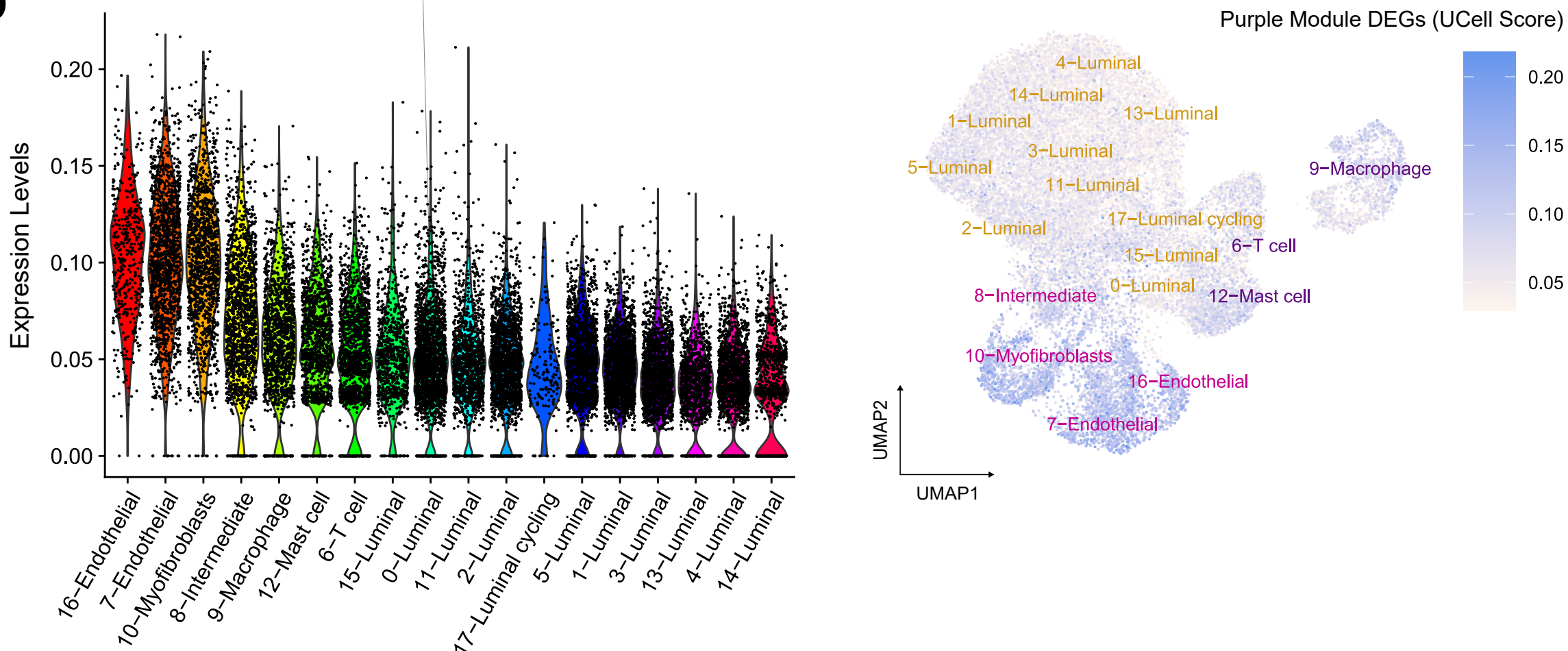

Supplement: Supplementary file 3 — Fig. S3. Functional enrichment and expression patterns of the purple module. [file MOL2-20-1429-s025.pdf]

**A**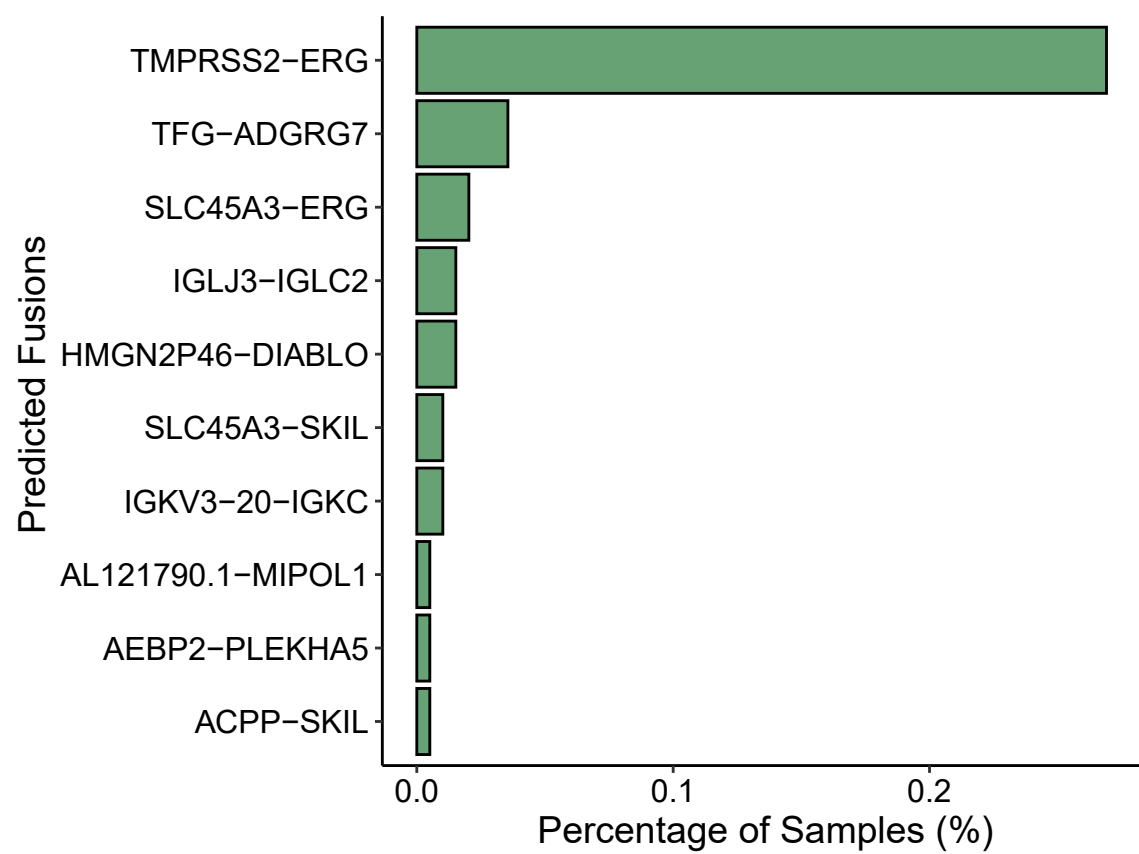**B**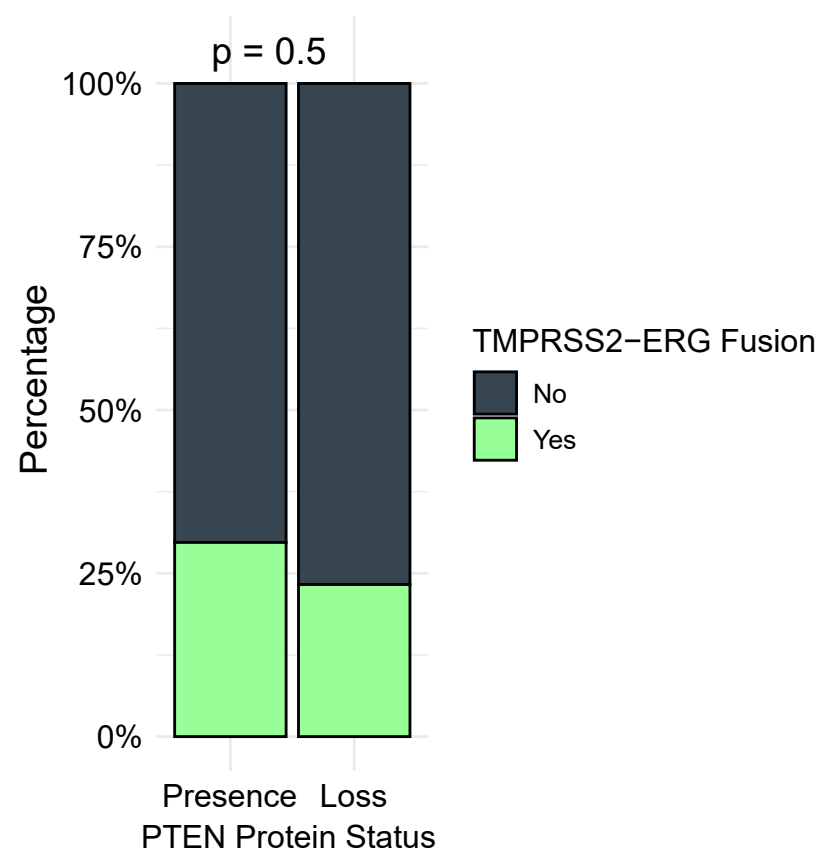**C**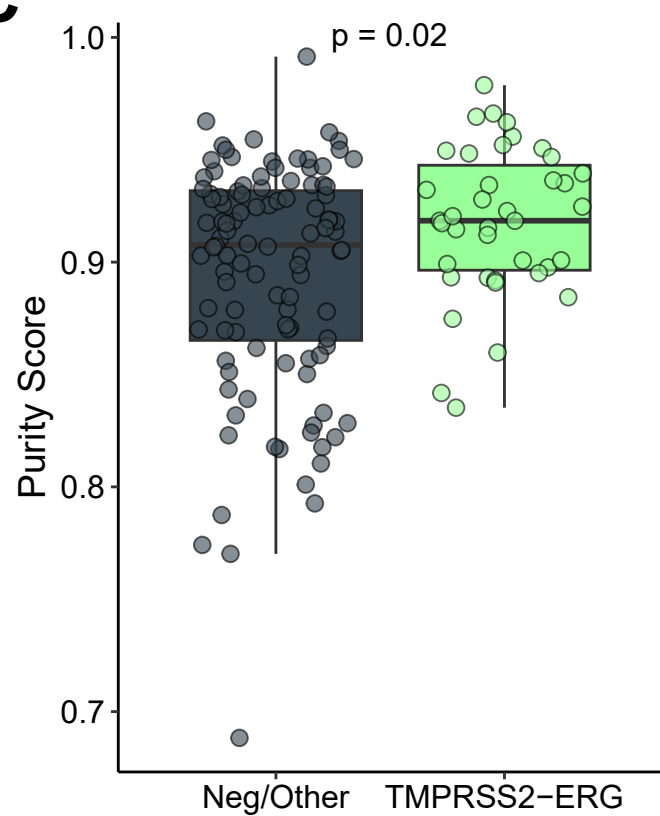**D**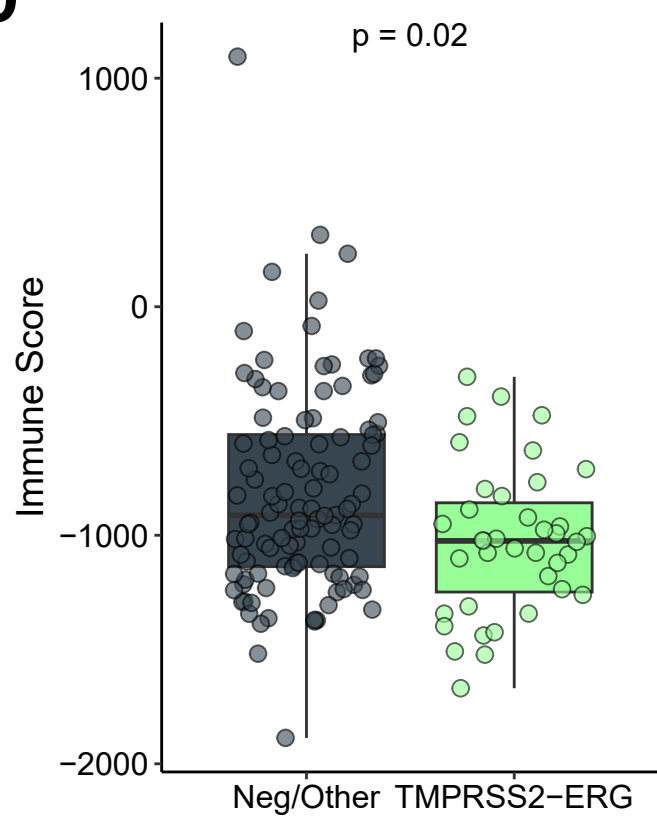**E**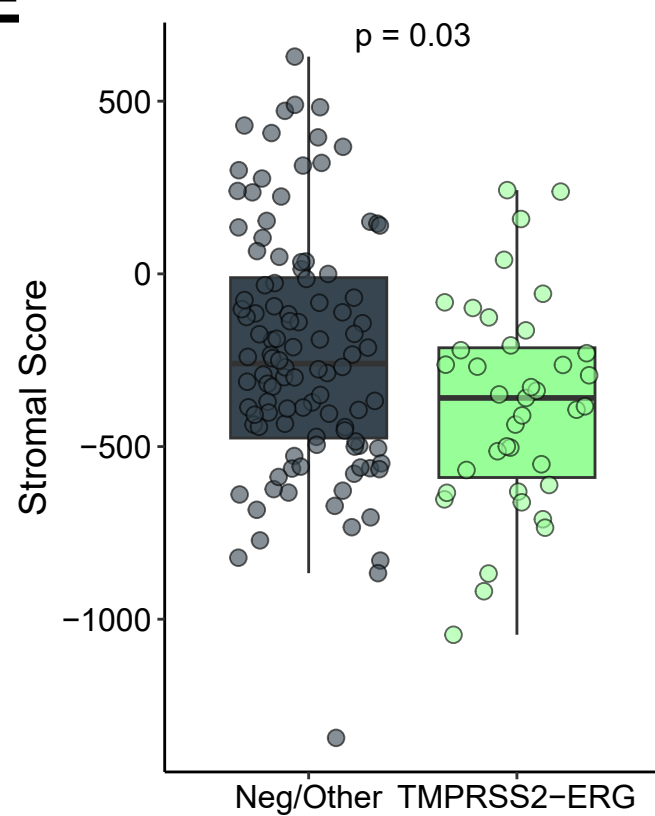**F**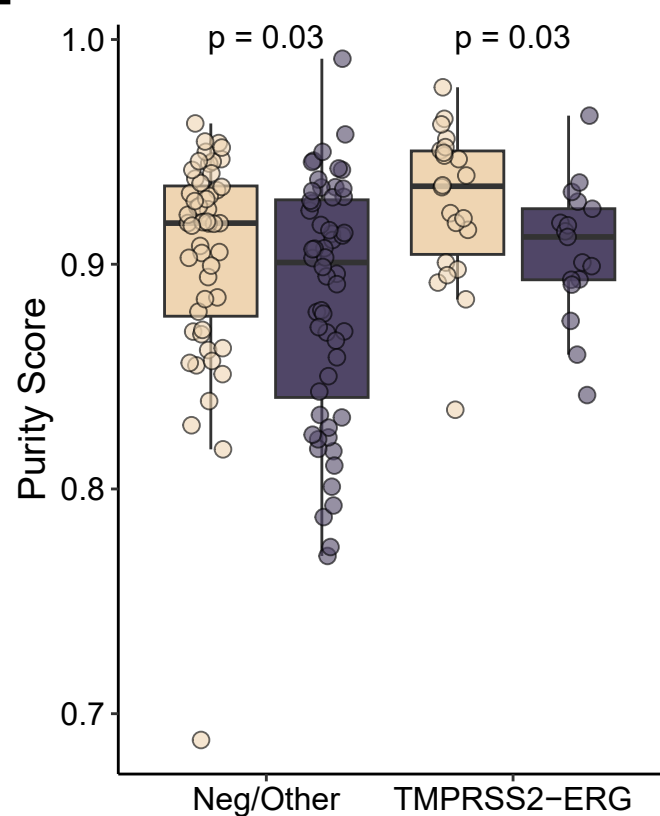**G**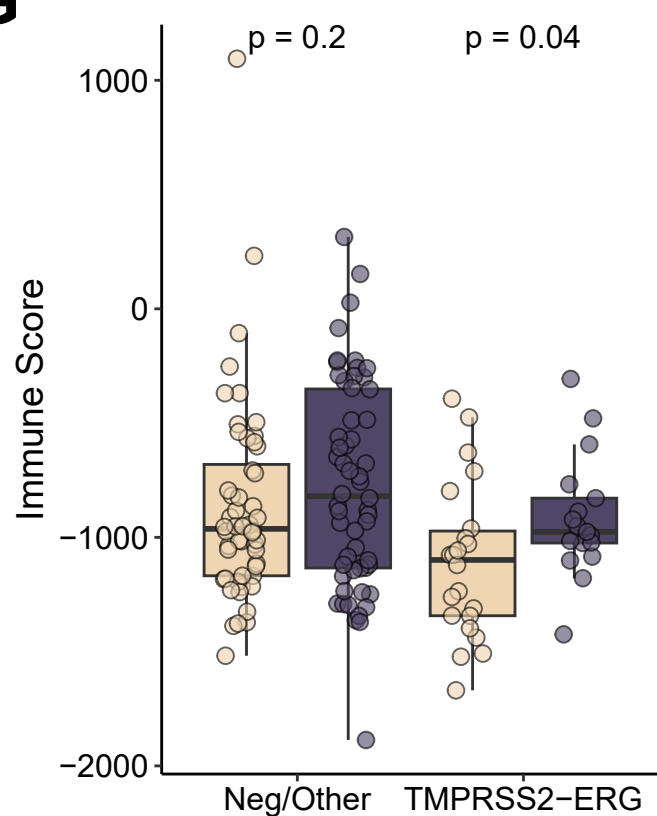**H**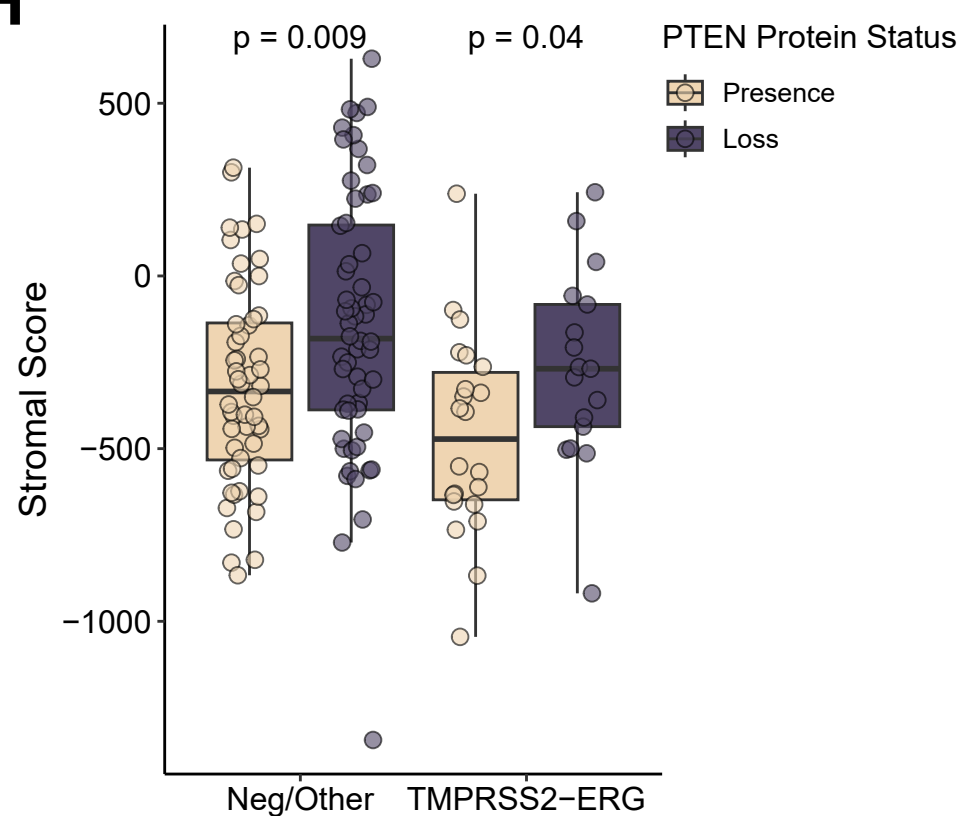**I**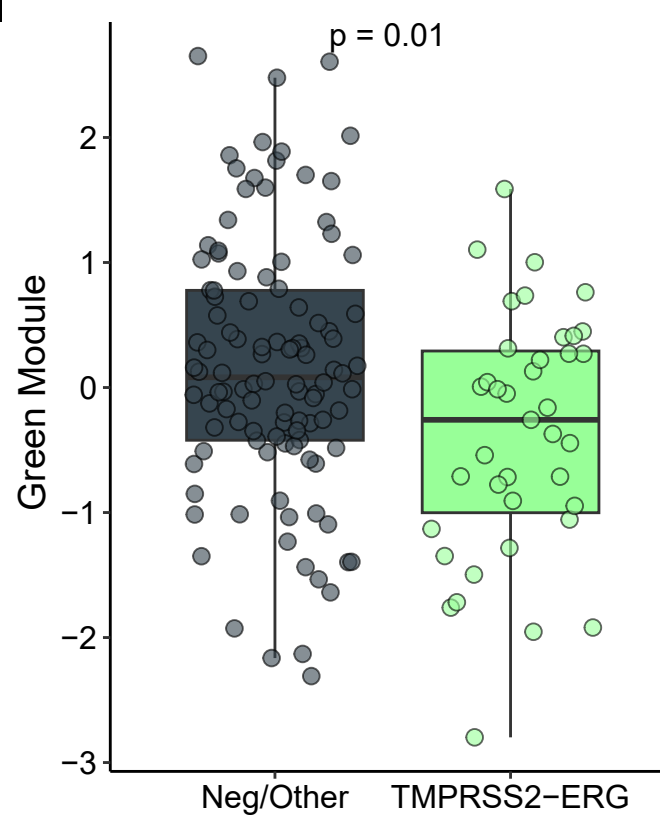**J**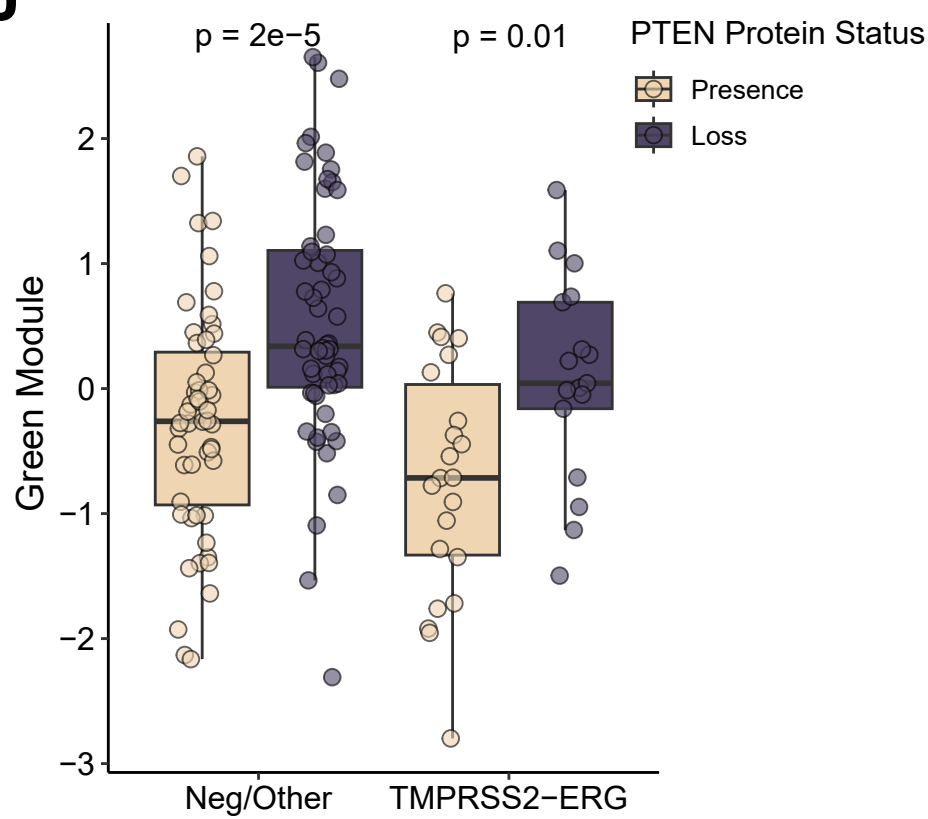

Supplement: Supplementary file 4 — Fig. S4. Association of ERG gene fusions with stromal infiltration and extracellular matrix (ECM) signature. [file MOL2-20-1429-s023.pdf]

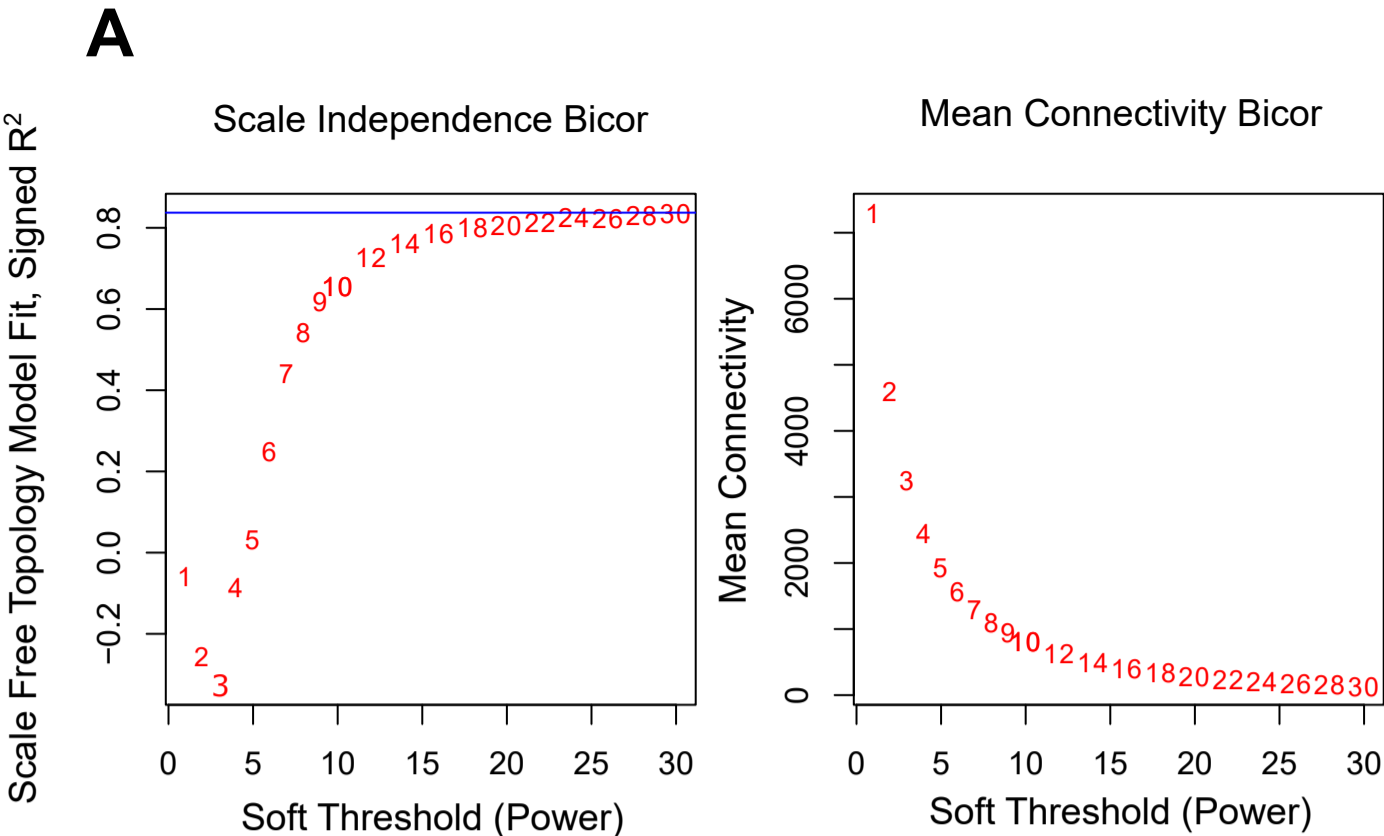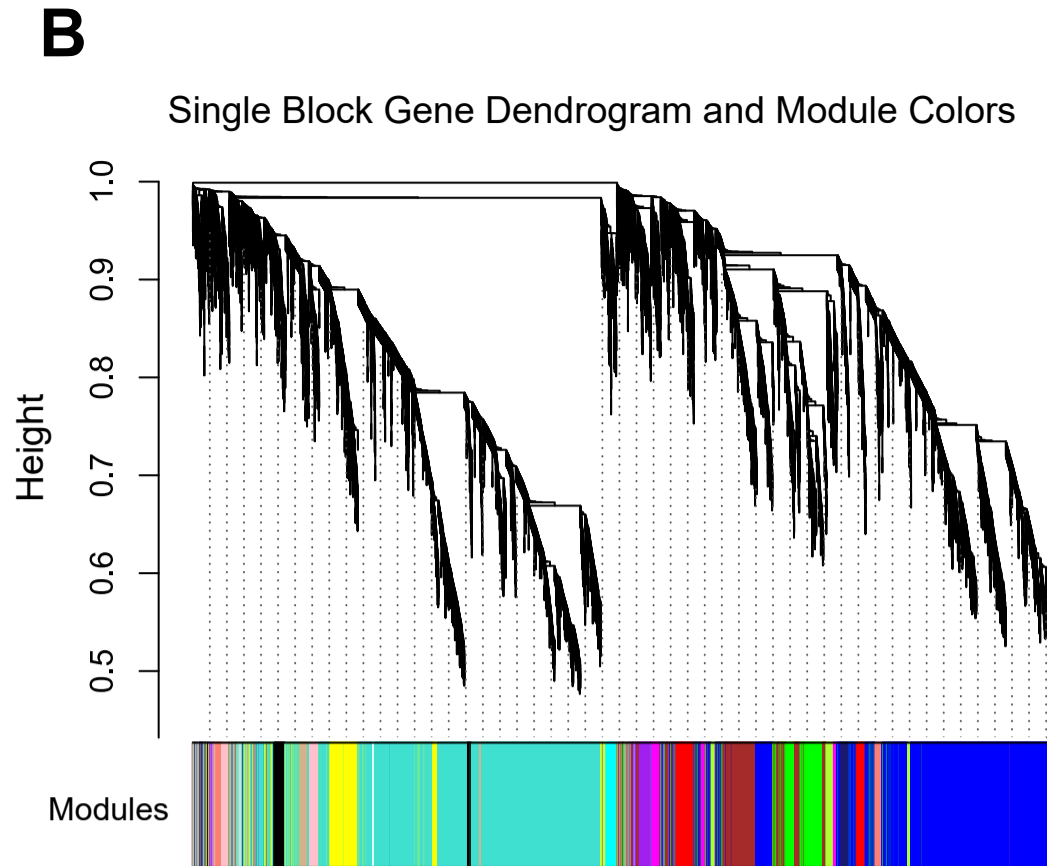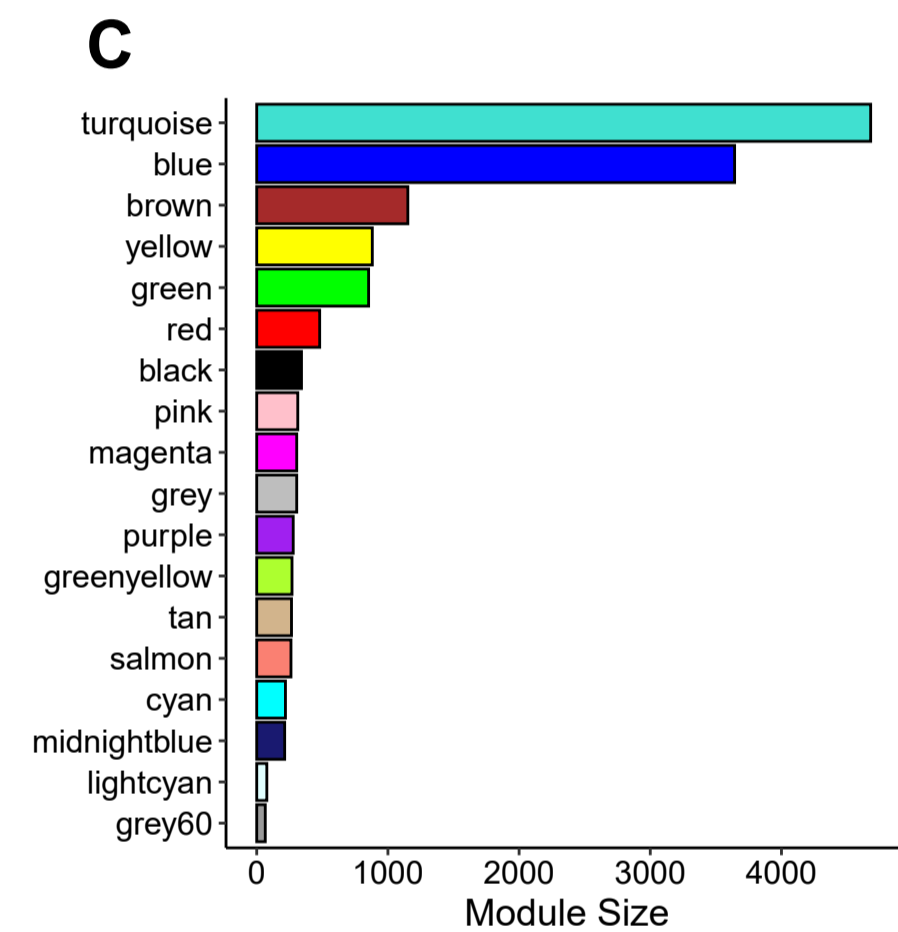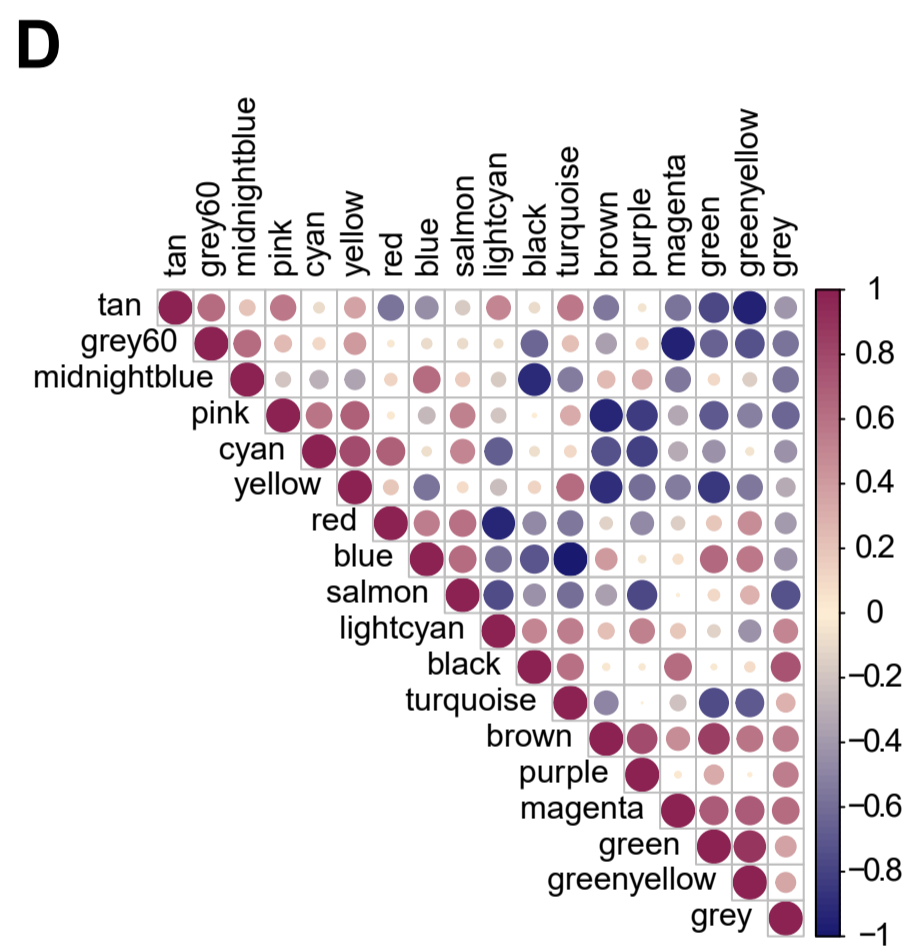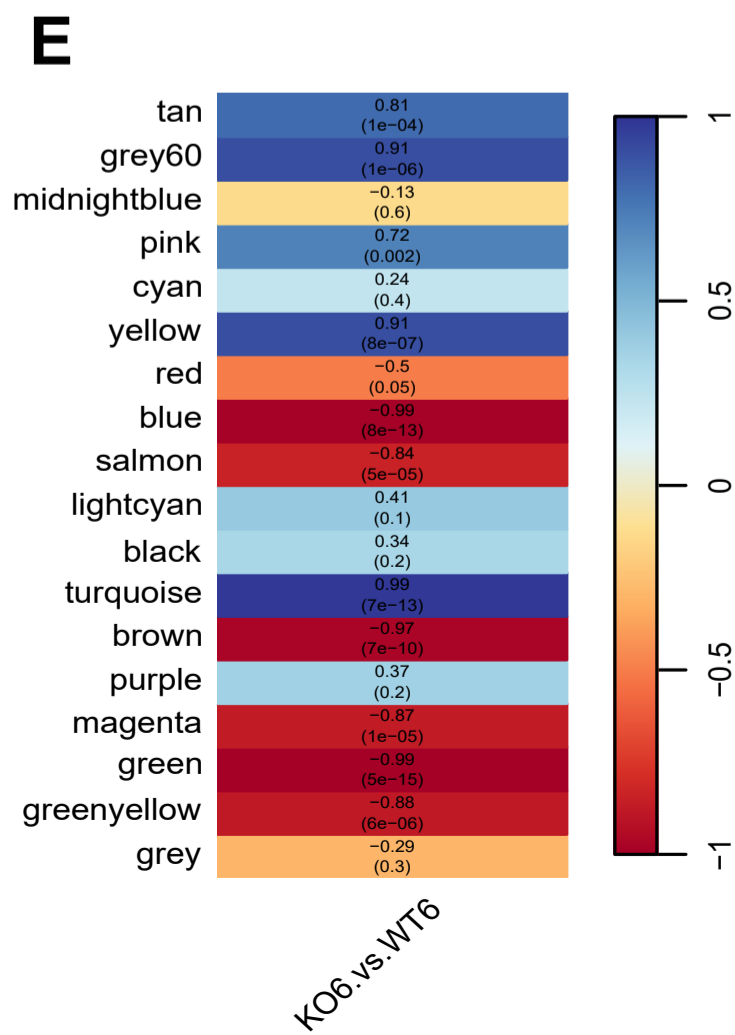

Supplement: Supplementary file 5 — Fig. S5. Gene network analyses using WGCNA in the mouse dataset. [file MOL2-20-1429-s010.pdf]

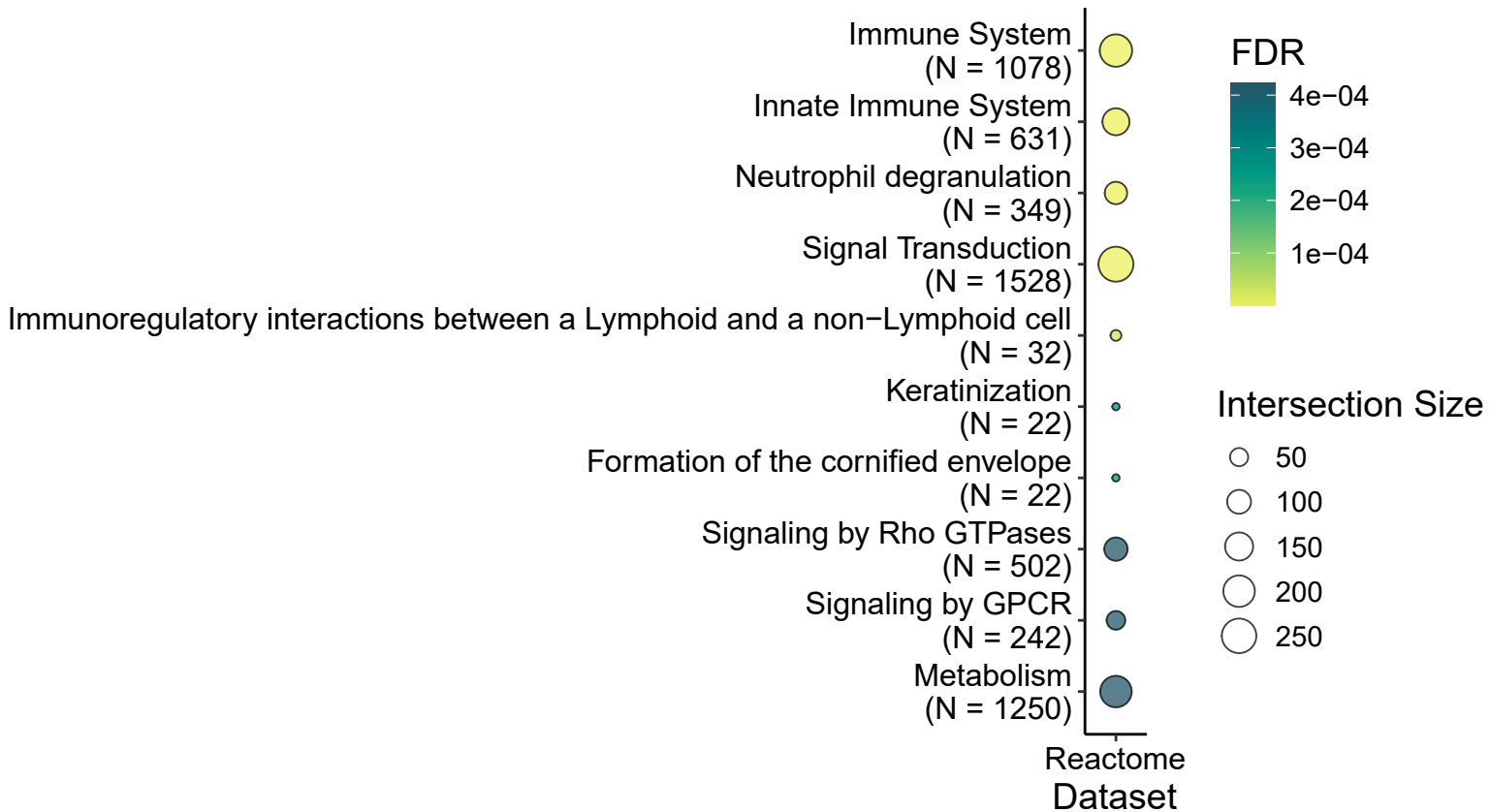

Supplement: Supplementary file 6 — Fig. S6. Functional enrichment analyses of the differentially expressed genes in the turquoise module. [file MOL2-20-1429-s013.pdf]

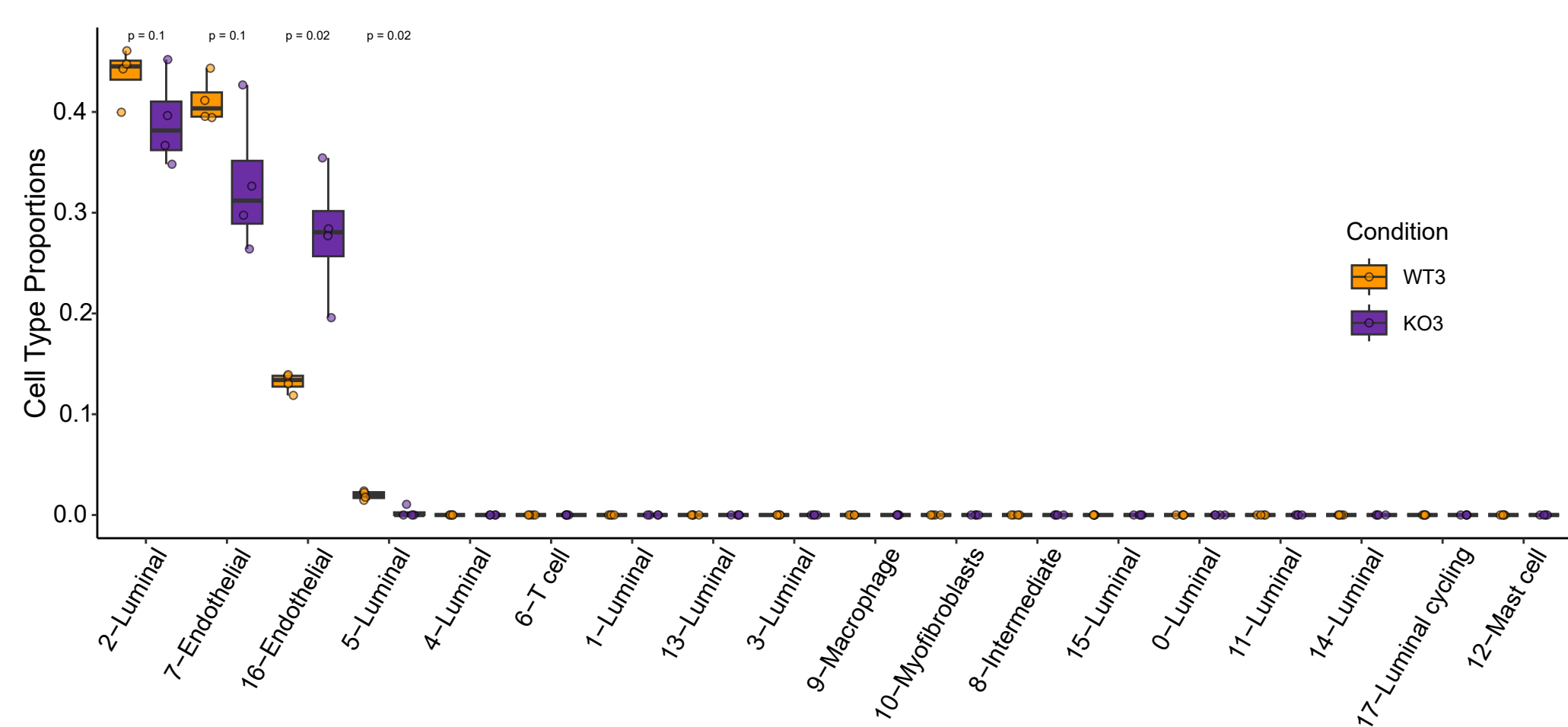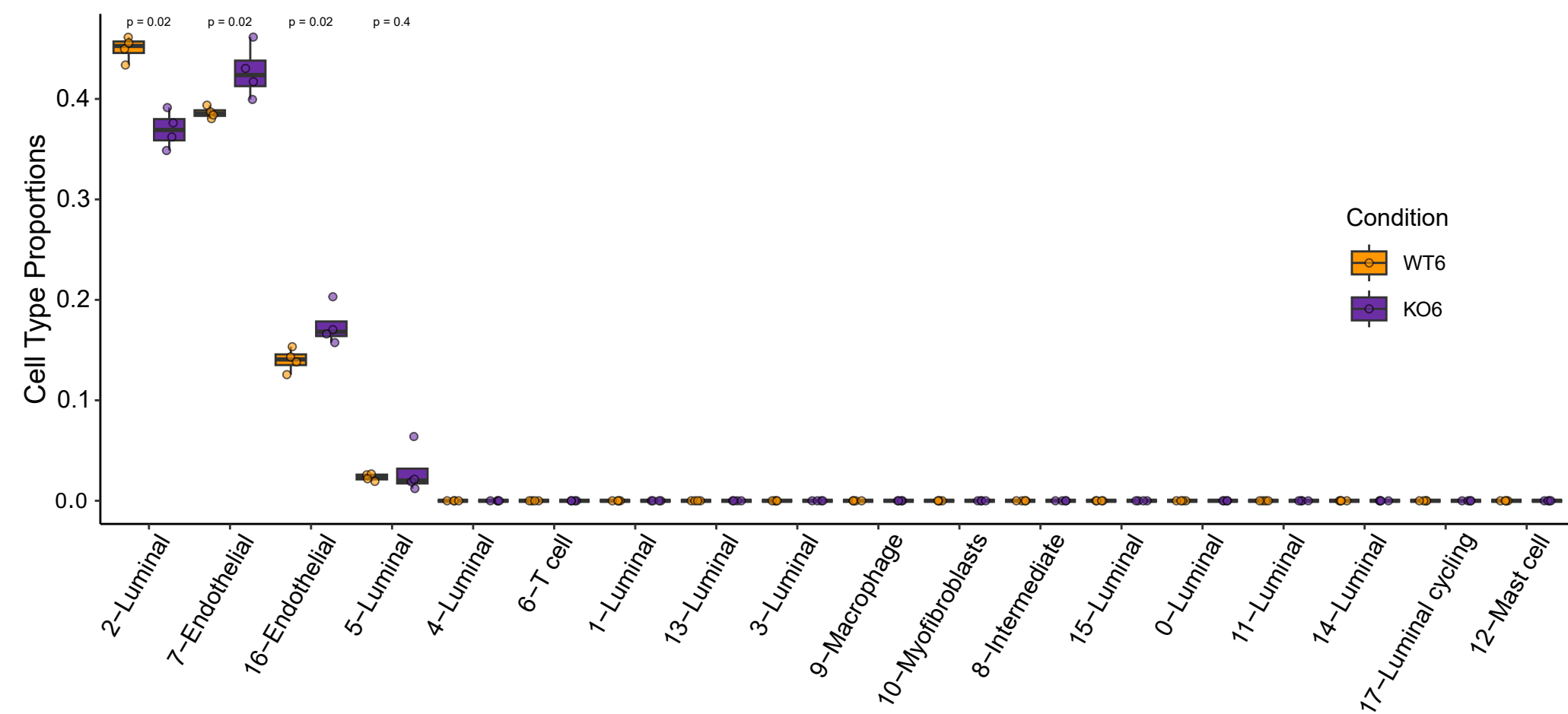

Supplement: Supplementary file 7 — Fig. S7. Deconvolution analysis of the mouse transcriptomic data using MuSiC. [file MOL2-20-1429-s022.pdf]

**A**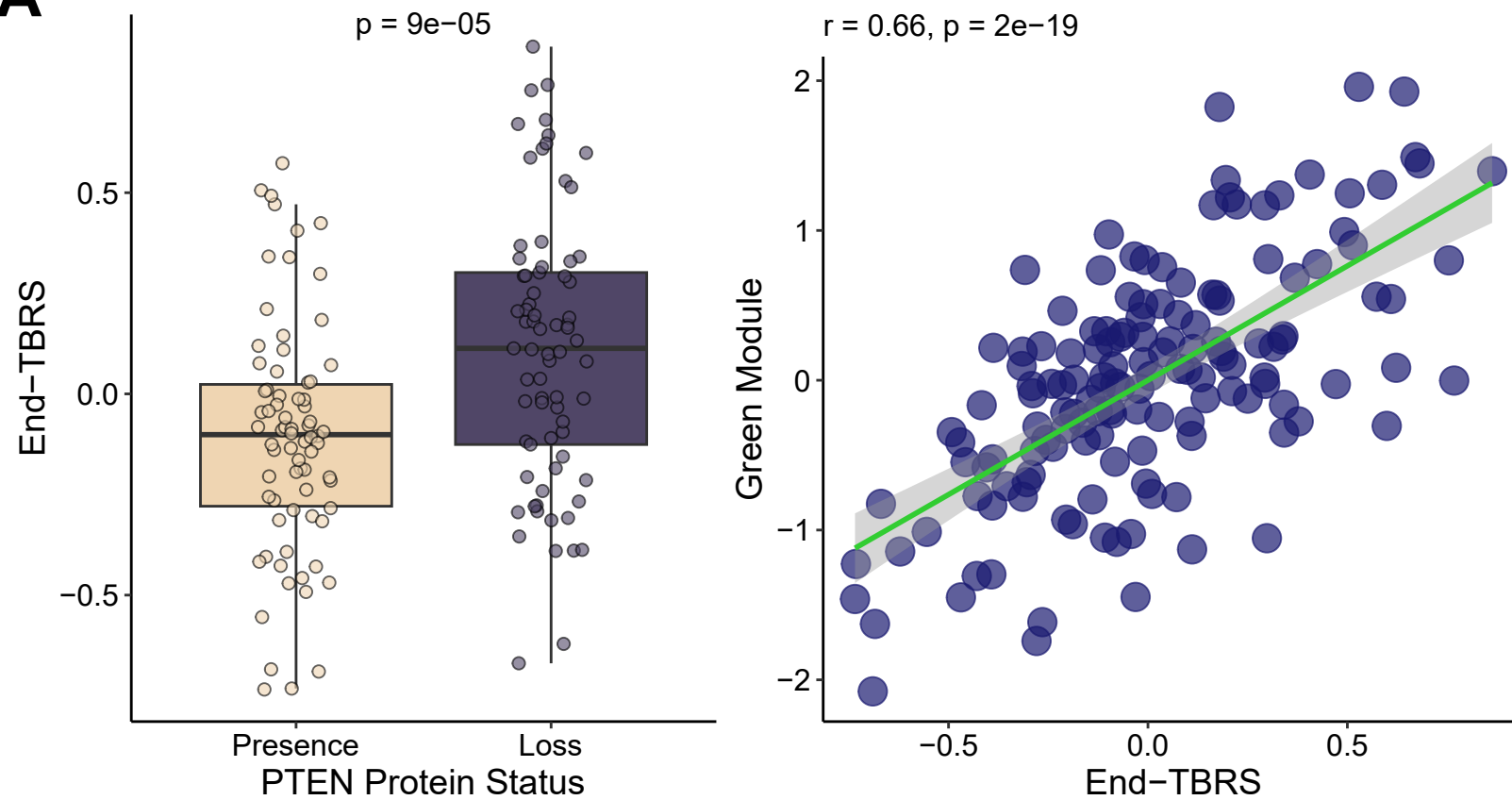**B**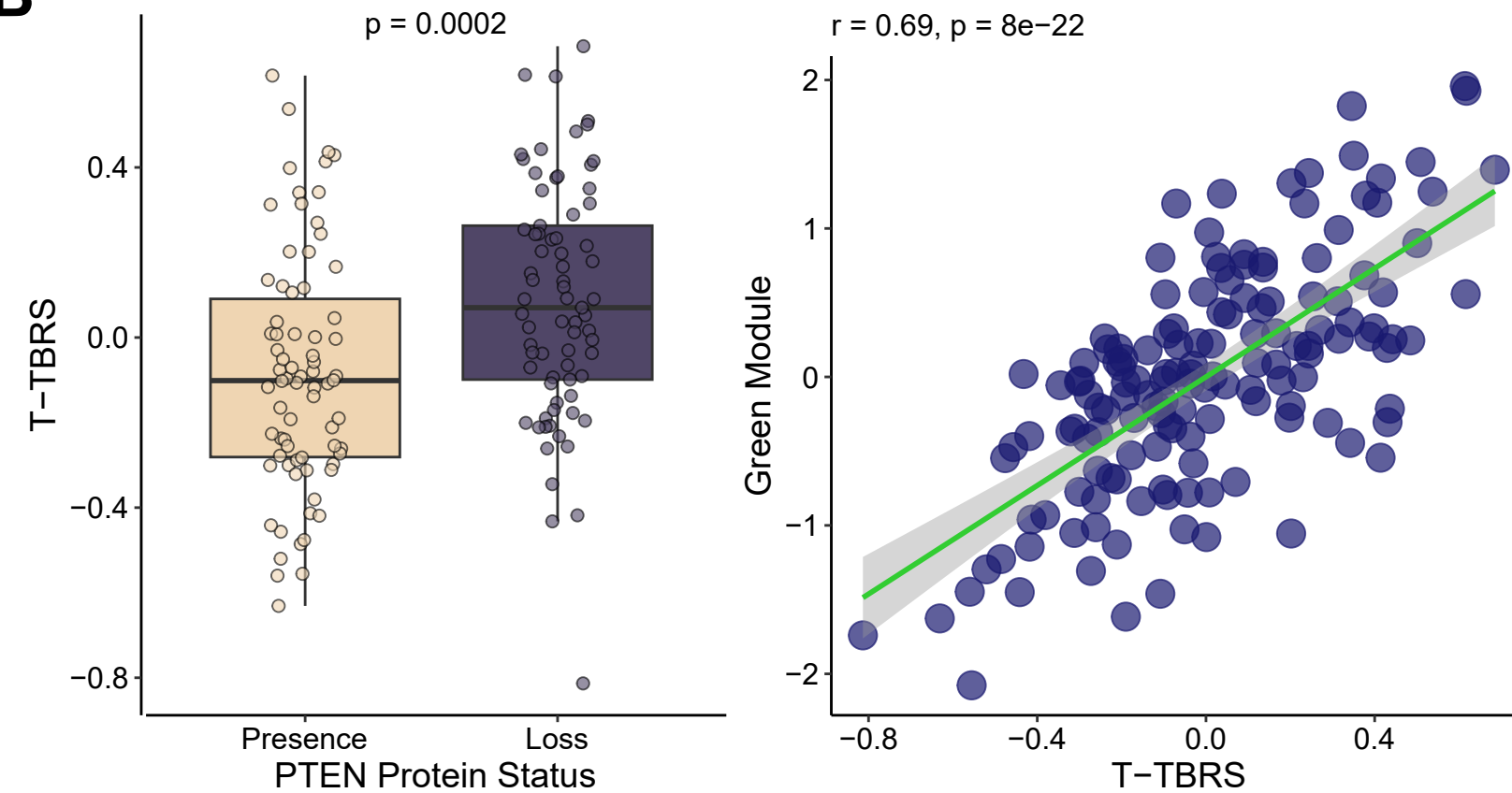**C**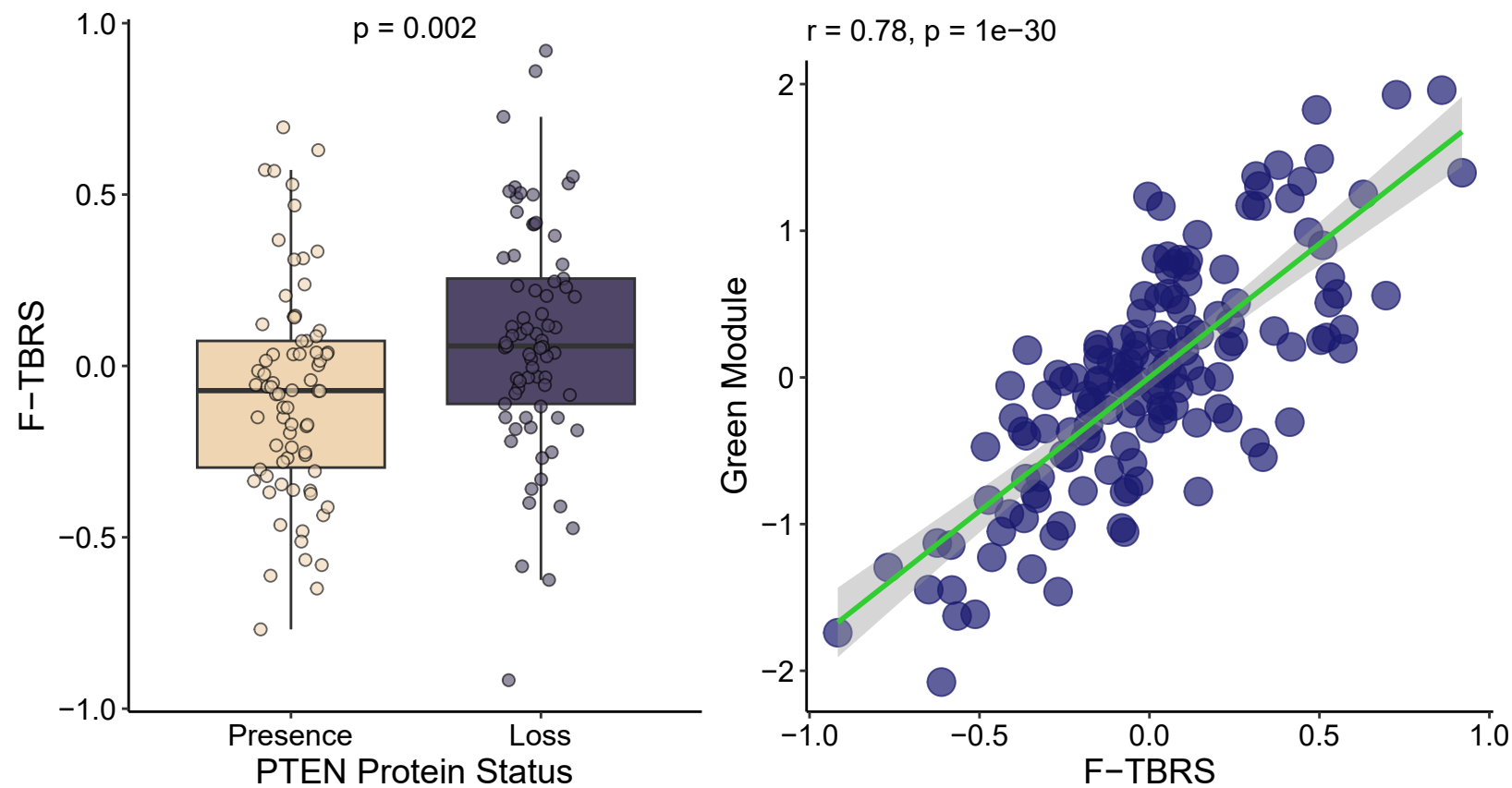**D**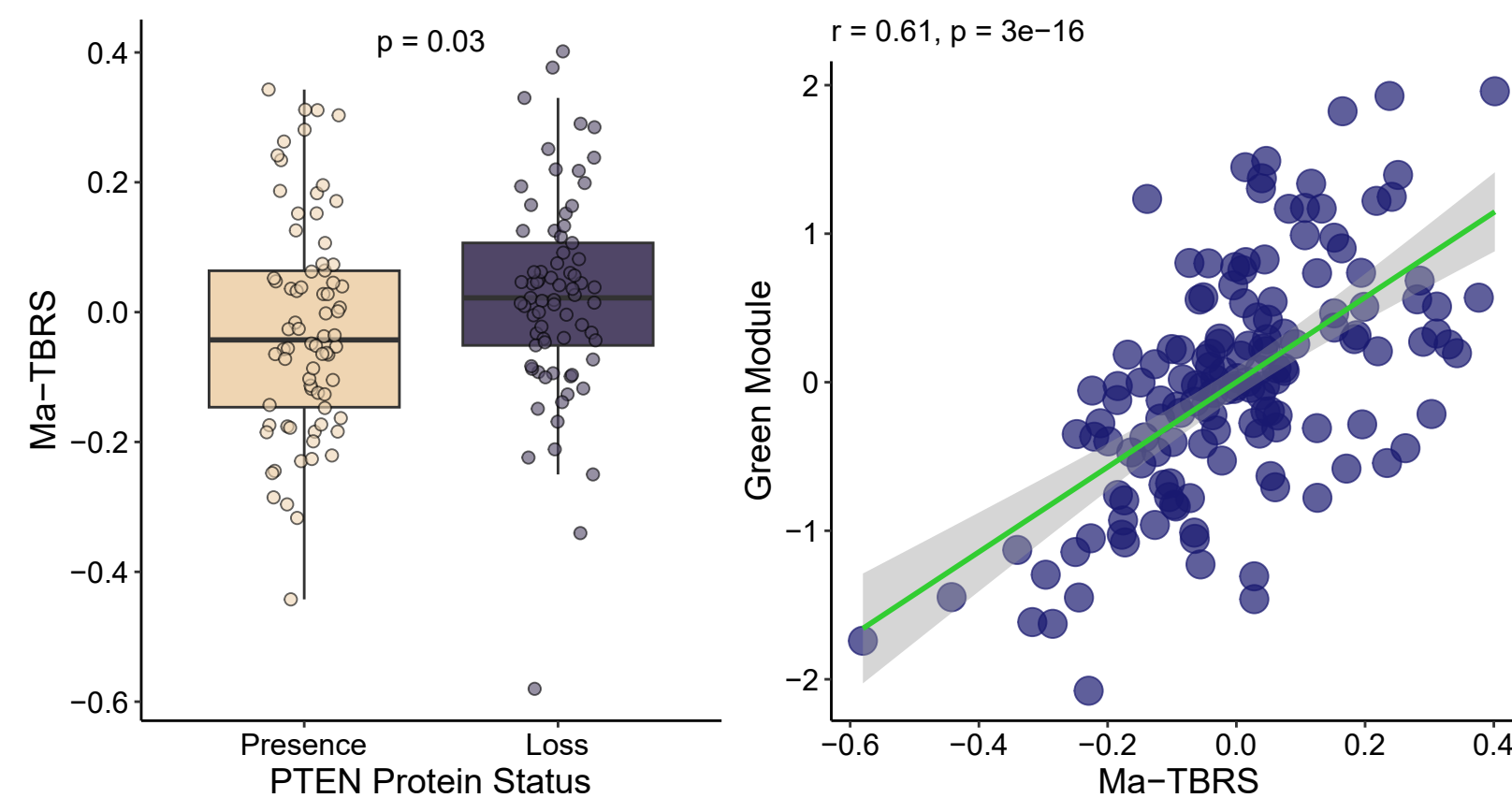

Supplement: Supplementary file 8 — Fig. S8. Loss of PTEN protein increases the transcriptional activity of the TGF‐β response signature (TBRS) across different cell types. [file MOL2-20-1429-s018.pdf]

# A Basurto

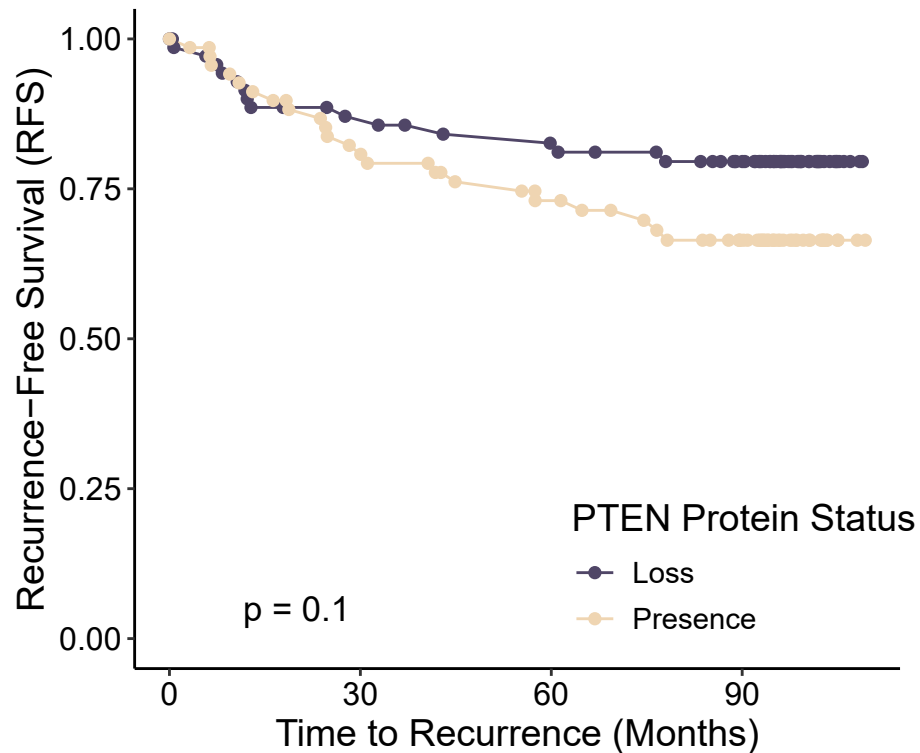

# B TCGA-PRAD

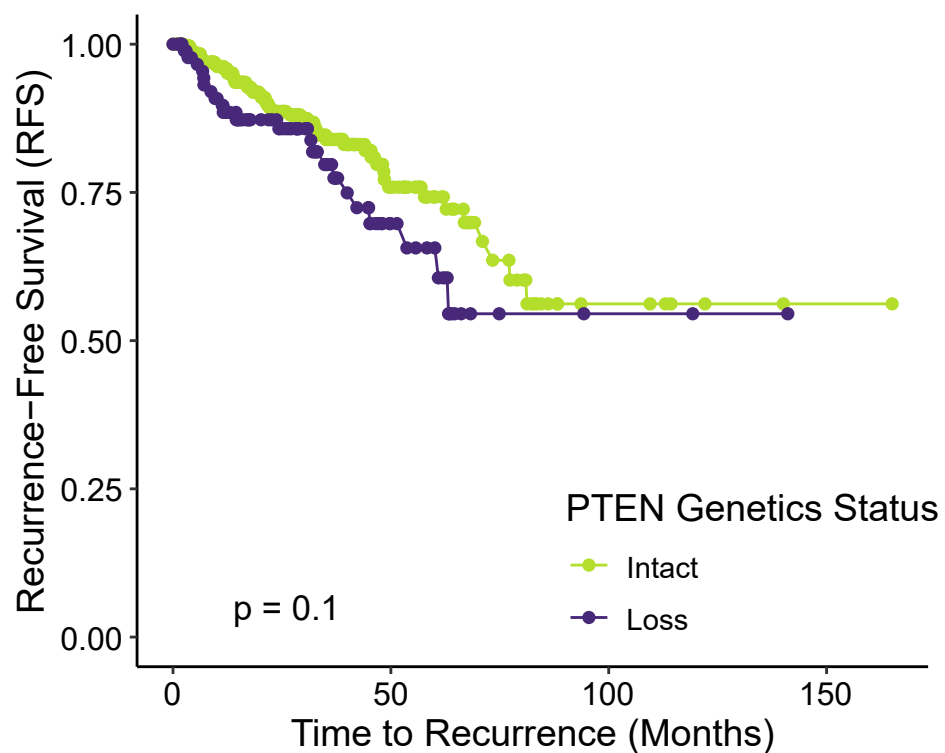

Supplement: Supplementary file 9 — Fig. S9. Recurrence‐free survival analyses with PTEN status. [file MOL2-20-1429-s007.pdf]

**A Basurto**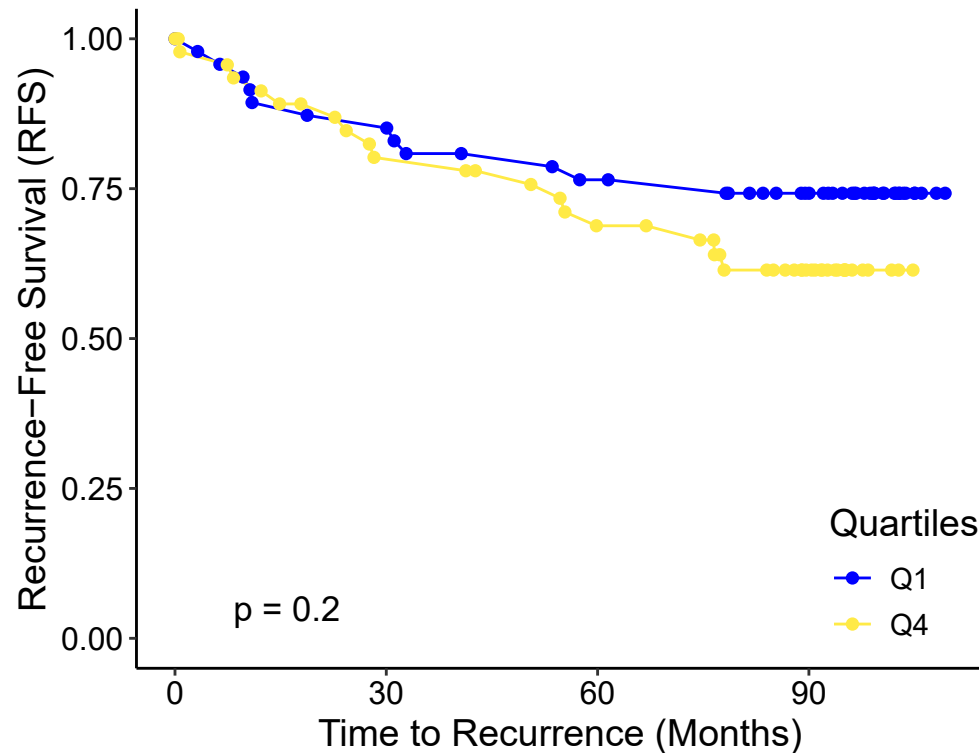**B TCGA-PRAD**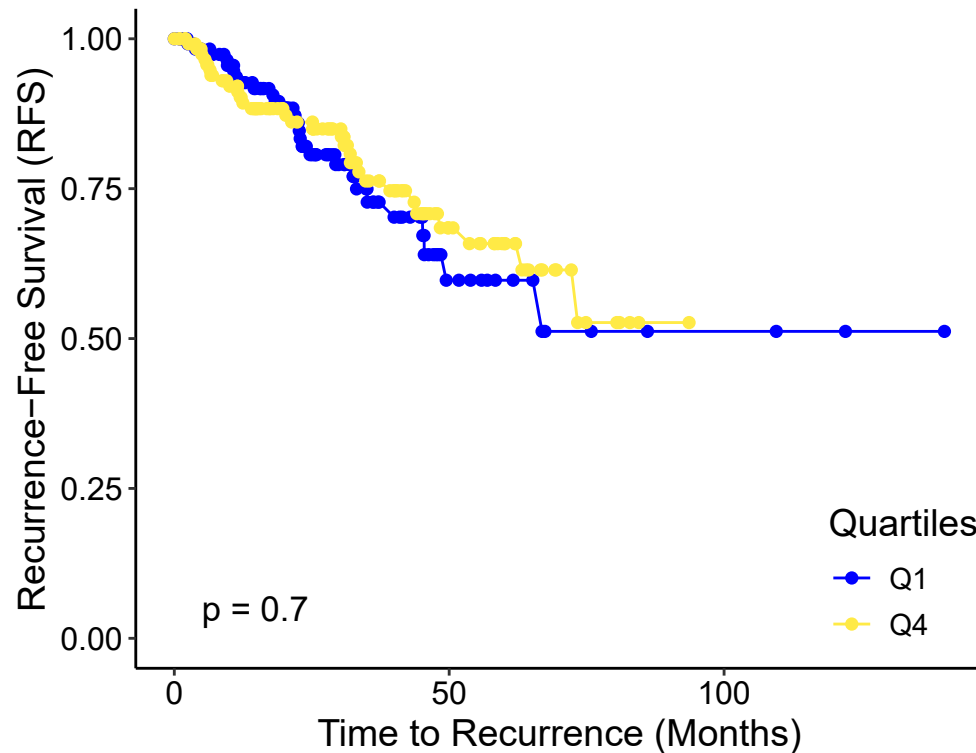

Supplement: Supplementary file 10 — Fig. S10. Recurrence‐free survival analyses with PI3K–AKT–mTOR signature. [file MOL2-20-1429-s009.pdf]
